# EIF1 coordinates transcriptomic and splicing networks associated with cell cycle dysregulation in diabetic retinopathy

**DOI:** 10.1371/journal.pone.0355473

**Published:** 2026-09-03

**Authors:** Na Li, Qian Ma, Xin Fan, Qi Liu, Li Liu, Shengfu Zhang, Yaling Ma, Hongxia Xu, Xinyi Li

**Affiliations:** 1 Department of Ophthalmology, General Hospital of Ningxia Medical University, Yinchuan, Ningxia Hui, China; 2 Beijing Tongren Hospital, Capital Medical University, Beijing, China; 3 Department of Pediatric Surgery, Peking University First Hospital Ningxia Women and Children’s Hospital (Ningxia Hui Autonomous Region Maternal and Child Health Hospital), Yinchuan, Ningxia Hui, China; 4 Department of Cardiovascular Surgery, General Hospital of Ningxia Medical University, Yinchuan, Ningxia Hui, China; 5 Medical Science and Technology Research Center, Ningxia Medical University, Yinchuan, Ningxia Hui, China; 6 DEYANG Ophthalmologic Hospital, Sichuan, China; Hirosaki University Graduate School of Medicine, JAPAN

## Abstract

**Purpose:**

EIF1, an RNA-binding protein implicated in multiple diseases, remains poorly characterized in diabetic retinopathy (DR). This study therefore investigated EIF1 expression and function in an in vitro model of DR pathogenesis.

**Methods:**

Human retinal pigment epithelial cells (ARPE-19) were exposed to 50 mM glucose to model DR. EIF1 was knocked down using siRNA under hyperglycemic conditions, followed by transcriptome sequencing (RNA-seq) to profile differentially expressed genes (DEGs) and alternative splicing events (ASEs). Key findings from RNA-seq were validated by RT-qPCR.

**Results:**

High-glucose treatment significantly suppressed cellular proliferation concurrent with EIF1 downregulation, suggesting that hyperglycemia-induced EIF1 depletion may impair retinal pigment epithelial cell functionality. Transcriptome sequencing revealed that EIF1 knockdown induced differential expression in 223 genes in total, comprising 35 upregulated and 188 downregulated genes. Notably, functional enrichment analysis revealed that downregulated genes are significantly enriched in cell cycle-related signaling pathways. Additionally, 1211 differential alternative splicing events were detected, primarily impacting cell cycle and p53 signaling pathways. Further analysis uncovered differentially expressed genes (e.g., BIRC5, MCM4, PLK1, RAD51, MELK, UBE2C, GINS2, and FANCA) and differentially spliced genes (e.g., COP1, TFE3) associated with the cell cycle after EIF1 knockdown. RT-qPCR validation confirmed the differential expression of key DEGs and the altered splicing patterns of COP1 and TFE3, consistent with RNA-seq predictions. These findings suggest that EIF1 may be associated with cell cycle dysregulation through the modulation of both gene expression and alternative splicing.

**Conclusions:**

This study suggests a potential role for EIF1 in DR pathogenesis in an in vitro model, identifying it as a candidate for further investigation. The data point to an association between EIF1 and the regulation of cell cycle-related genes at both the transcriptional and splicing levels.

## 1 Introduction

Diabetic retinopathy (DR) represents one of the most prevalent microvascular complications of diabetes, affecting 30% to 40% of diabetic patients [1,2]. With over 100 million individuals affected globally, DR has emerged as a leading cause of blindness and visual impairment [3,4]. The rising prevalence of diabetes has led to a corresponding increase in DR incidence, placing substantial socioeconomic burdens on healthcare systems worldwide [5]. Although various treatment strategies are currently available to delay DR progression, its exact molecular mechanisms have not been fully elucidated, limiting the development of more effective treatments. In recent years, advances in high-throughput transcriptome sequencing technology have enabled us to explore the molecular regulatory networks associated with DR, providing new directions for early disease diagnosis and targeted therapy [6].

Eukaryotic translation initiation factor 1 (EIF1; aliases: A121, ISO1, SUI1, EIF-1, EIF1A), a 12-kDa protein that functions as a critical mediator of start codon recognition during eukaryotic translation initiation [7,8]. Beyond its canonical role in translation initiation [9], EIF1 has been classified as an RNA-binding protein (RBP) based on its ability to interact with RNA molecules, and emerging evidence suggests that it may participate in broader regulatory processes, including alternative splicing and cell cycle control, although these functions remain poorly characterized in the context of DR [10,11]. Nevertheless, the mechanistic underpinnings of EIF1’s RBP functionality in DR remain enigmatic.

In this study, we employed RNA sequencing technology to investigate the functional role of EIF1 in ARPE-19 cells under high-glucose conditions, as well as its regulatory impact on genome-wide transcriptional dynamics and alternative splicing profiles. By integrating analyses of differential gene expression and splicing variation patterns, we aim to explore potential molecular mechanisms through which EIF1 may be involved in the pathological progression of DR, potentially through its effects on RNA expression levels and alternative splicing events, while identifying candidate downstream effectors and biological pathways within its regulatory network. This research may contribute to the understanding of DR pathogenesis by suggesting potential connections between EIF1-mediated transcriptional and post-transcriptional regulatory networks, and offers a foundation for further investigation into potential therapeutic strategies..

## 2 Materials and methods

### 2.1 Cloning and plasmid construction

All siRNAs were purchased from Gemma (Suzhou, China). Non-targeting control siRNA (siNegative): 5’-UUCUCCGAACGUGUCACGUTT-3’ (sense). siRNA targeting EIF1 (siEIF1): 5’- GAAACUAGUGAAGGCGUUUTT -3’ (sense).

### 2.2 Cell culture and transfections

The ARPE-19 cells line (CL-0026 Procell Life Science & Technology Co., Ltd., China) were cultured at 37℃ with 5% CO_2_ in DMEM/F12 (PM150312, Procell Life Science & Technology Co., Ltd., China) with 10% fetal bovine serum (FBS) (10099-141, Gibco, Invitrogen), 100 µg/mL streptomycin, 100 U/mL penicillin. siRNA transfection of ARPE-19 cells was performed using Lipofectamine™ RNAiMAX Transfection Reagent Transfection Reagent (Invitrogen, Carlsbad, CA, USA) according to the manufacturer’s protocol. Transfected cells were harvested after 48 h for RT-qPCR and western blotting analysis.

### 2.3 Cell proliferation assay

The cell proliferation assay was conducted using a Cell Counting kit-8 (CCK-8, HY-K0301, MCE, Shanghai, China). Briefly, ARPE-19 cells were seeded in 24-well plates at a density of 20,000 cells/well and divided into three groups: experimental groups treated with 30 mM or 50 mM glucose and normal controls containing culture medium only (5.5 mM glucose). Following cultured for 48 h, 10 μL CCK-8 solution was added to the culture medium and incubated for an additional 3 h. Subsequent to this, the optical density of the cells was measured with a Microplate Reader (ELX800, Biotek, USA) at an absorbance of 450 nm. The net cell proliferation rate was calculated using the following formula: proliferation rate = (experimental OD value − blank OD value) / (control OD value − blank OD value) × 100%.

### 2.4 RT-qPCR

To assess the expression level of EIF1 and other target genes, RT-qPCR was performed with GAPDH (glyceraldehyde-3-phosphate dehydrogenase) as the control gene. Reactions were run on an ABI QuantStudio 5 instrument under the following conditions: 95°C for 10 min, followed by 40 cycles of 95°C for 15 s and 60°C for 1 min. Each sample was tested in triplicate as technical replicates. The concentration of each transcript was then normalized to GAPDH mRNA level using 2^-ΔΔCT^ method [11]. To quantitatively analyze the two different splicing isoforms of a specific ASE, primers complementary to the splice junction of the constitutive exon and alternative exon were designed to specifically amplify each of the two isoforms. Comparisons were performed with the paired Student’s t-test by using GraphPad Prism software (San Diego, CA). Primer sequences and amplicon lengths for all qPCR and ASE validation experiments are provided in Supplementary S1 Table.

### 2.5 Western blot

ARPE-19 cells were lysed in ice-cold RIPA Buffer (PR20001, Proteintech, China) supplemented with a protease inhibitor cocktail (4693116001, sigma, USA) and incubate on ice for 30 min. Samples were boiled for 10 min in boiling water with protein loading buffer (P1040, Solarbio, China) and loaded onto 10% SDS-PAGE gel and transferred onto 0.45 mm PVDF membranes (ISEQ00010, Millipore, USA). The PVDF membranes were then blocked for 1h at room temprature and incubated overnight at 4℃ with primary antibody respectively against EIF1 (anti-EIF1, 1:1000, antibody produced in rabbit, 15276-1-AP, Proteintech, China) and GAPDH (1:10000, antibody produced in mouse, 60004-1-lg, Proteintech, China), followed by an incubation with horseradish peroxidase-conjugated secondary antibody (anti-rabbit, 1:10000, SA00001-2, Proteintech, China or anti-mouse, 1:10000, AS003, ABclonal, China) for 45 min at room temprature. Then, membranes were visualized using the enhanced ECL reagent (P0018FM, Beyotime, China) through chemiluminescence. Protein bands were quantified by densitometry using ImageJ software (NIH, USA), and band intensities were normalized to GAPDH.

### 2.6 RNA extraction and sequencing

Total RNA was treated with RQ1 DNase (Promega) to remove DNA. The quality and quantity of the purified RNA were determined by measuring the absorbance at 260 nm/280 nm (A260/A280) using smartspec plus (BioRad). RNA integrity was further verified by 1.5% agarose gel electrophoresis.

For each sample, 1 μg of total RNA was treated with RQ1 DNase (Promega) to remove DNA before used for directional RNA-seq library preparation by VAHTS® Universal V8 RNA-seq Library Prep Kit for Illumina (N605) for RNA-seq library preparation. mRNAs were captured by VAHTS mRNA capture Beads (Vazyme, N401). Fragmented mRNAs were converted into double strand cDNA. Following end repair and A tailing, the DNAs were ligated to VAHTS RNA Multiplex Oligos Set 1 for Illumina (N323), the ligated products were amplified, purified, quantified and stored at −80℃ before sequencing. The strand marked with dUTP (the 2nd cDNA strand) is not amplified, allowing strand-specific sequencing.

Three independent biological replicates were prepared for each condition (si-NC and si-EIF1). All six samples were processed in parallel for library preparation and sequenced in a single run to minimize batch effects. For high-throughput sequencing, the libraries were prepared following the manufacturer’s instructions and applied to Illumina Novaseq 6000 system for 150 nt paired-end sequencing.

### 2.7 RNA-Seq filtering and differentially expressed genes (DEG) analysis

Raw reads containing more than 2-N bases were first discarded. Then adaptors and low-quality bases were trimmed from raw sequencing reads using FASTX-Toolkit (Version 0.0.13). The short reads less than 16nt were also dropped. After that, clean reads were aligned to the GRCh38 genome by HISAT2 [12] allowing 4 mismatches. For visualization purposes, uniquely mapped reads were used for gene reads number counting and FPKM calculation (fragments per kilobase of transcript per million fragments mapped) [13].

All differential expression analysis was performed using DESeq2 [14], which internally applies its own normalization procedure based on the median of ratios method, directly on the raw count data to ensure statistical rigor. The P value for correction <0.05 and fold change>2 or < 0.5 were set as the cut-off criteria for identifying DEGs.

### 2.8 Alternative splicing analysis

The alternative splicing events (ASEs) and regulated alternative splicing events (RASEs) between the samples were defined and quantified by using the ABLas (v0.2) pipeline as described previously [15,16]. In brief, ABLas detection of ten types of ASEs was based on the splice junction reads, including exon skipping (ES), alternative 5’ splice site (A5SS), alternative 3’splice site (A3SS), mutually exclusive exons (MXE), mutually exclusive 5’UTRs (5pMXE), mutually exclusive 3’UTRs (3pMXE), cassette exon, A3SS&ES and A5SS&ES. For sample pair comparison, Fisher’s exact test was selected to determine statistical significance, using the alternative reads and model reads of the samples as input data. We calculated the changed ratio of alternatively spliced reads and constitutively spliced reads between compared samples, which was defined as the RASE ratio. The RASE ratio >=0.2 and p-value <= 0.05 were set as the threshold for RASEs detection. For repetition comparison, Student’s t-test was performed to evaluate the significance of the ratio alteration of AS events. Those events which were significant at P-value cutoff of 0.05 were considered non intron retention (NIR) RASEs.

To assess RBP regulated ASE, Student’s t-test was performed to evaluate the significance of the ratio alteration of AS events. Those events which were significant at P-value cutoff corresponding to a false discovery rate cutoff of 5% were considered RBP regulated ASEs.

### 2.9 Functional enrichment analysis

To sort out functional categories of DEGs, Gene Ontology (GO) terms and KEGG pathways were identified using KOBAS 2.0 server [17]. Hyper geometric test and Benjamini-Hochberg FDR controlling procedure were used to define the enrichment of each term.

This gene dataset was subjected to pathway and process enrichment analysis with the Metascape bioinformatics online tool (www.metascape.org) that uses several ontology sources: KEGG Pathway, GO Biological Processes, Reactome Gene Sets, Canonical Pathways, CORUM, and WikiPathways.

### 2.10 Statistical analysis

The data from at least three independent experiments were presented as mean ± standard deviation (SD) for cellular experimental data, and as mean ± standard error of the mean (SEM) for RNA-seq derived expression values (e.g., FPKM bar plots). Comparisons between two groups were performed using two-tailed Student’s t-test. For multiple-group comparisons, two-way ANOVA followed by Tukey’s multiple comparisons test was applied. A p-value <0.05 was considered statistically significant (*p < 0.05; **p < 0.01; ***p < 0.001). All graphs were made using the GraphPad Prism software (Version number8.0, San Diego, CA).

## 3 Results

### 3.1 High glucose suppresses EIF1 expression in ARPE-19 cells

**Fig 1:**
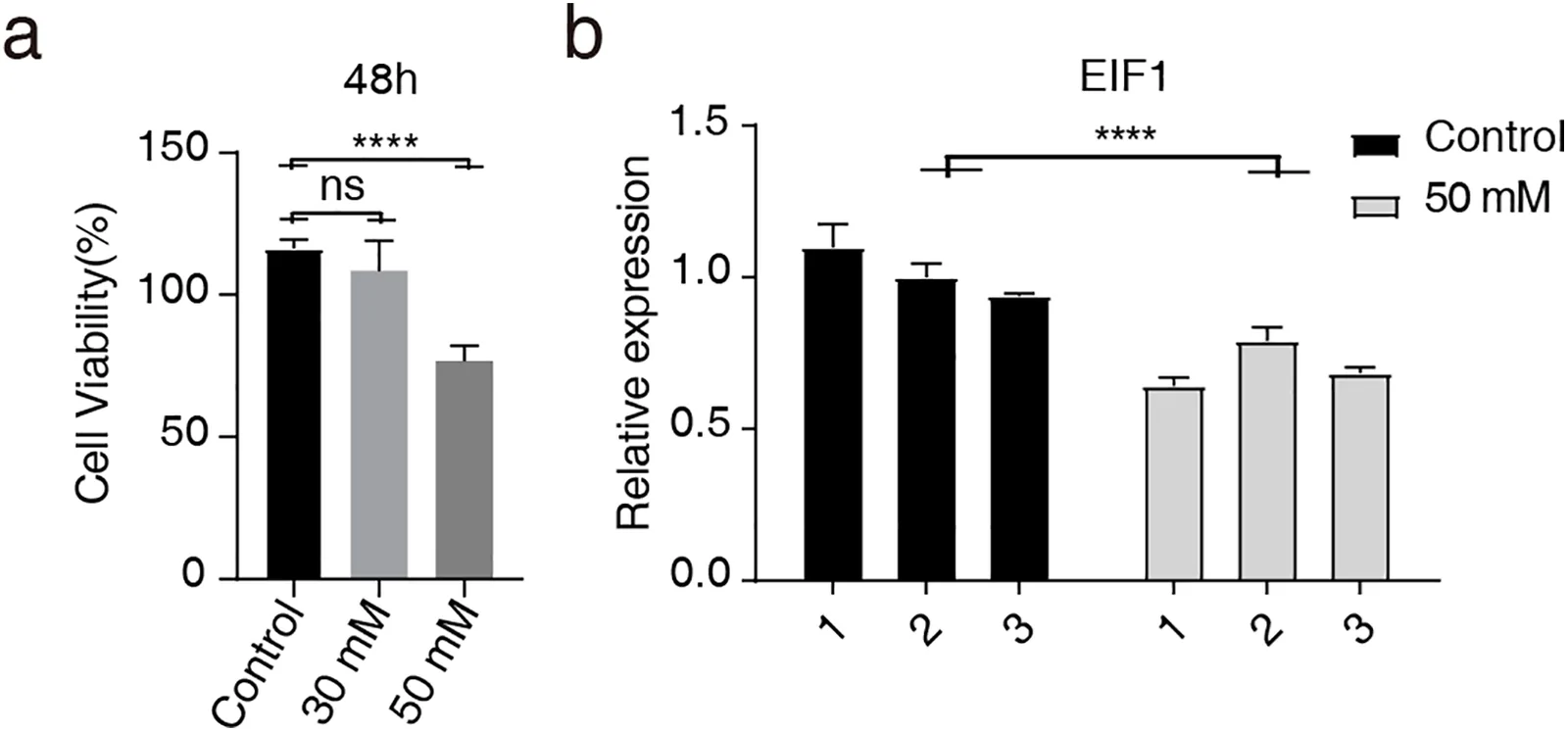
High glucose suppresses EIF1 expression in ARPE-19 cells. a. ARPE-19 cells are treated with indicated concentrations of glucose (0, 30, and 50 mM) for 48 hours.. Cell proliferation was assessed using the CCK-8 assay. Data are shown as mean ± SD (n = 3 per group). **** p-value < 0.0001 vs 0 mM control. b. The histogram showed the RT-qPCR results of 0 mM and 50 mM high glucose solution. Data are shown as mean ± SD (n = 3 per group). **** p-value < 0.0001.

To establish an in vitro DR model, this study stimulated human retinal pigment epithelial cells (ARPE-19) with gradient concentrations of glucose (0, 30 and 50 mM). The CCK-8 cell viability assay revealed a significant reduction in net proliferation rates in the 50 mM glucose-treated group compared to controls (****p < 0.0001), thus selecting this concentration for subsequent high-glucose interventions (Fig 1a). Further analysis via RT-qPCR demonstrated that mRNA expression levels of EIF1 in the high-glucose-treated group were markedly downregulated relative to the normal control group (***p < 0.001) (Fig 1b). These findings suggest that exposure to high glucose conditions may impair retinal pigment epithelial cell function by suppressing EIF1 expression.

### 3.2 EIF1 depletion impairs retinal pigment epithelial cell function via cell cycle gene dysregulation in hyperglycemic models

**Fig 2:**
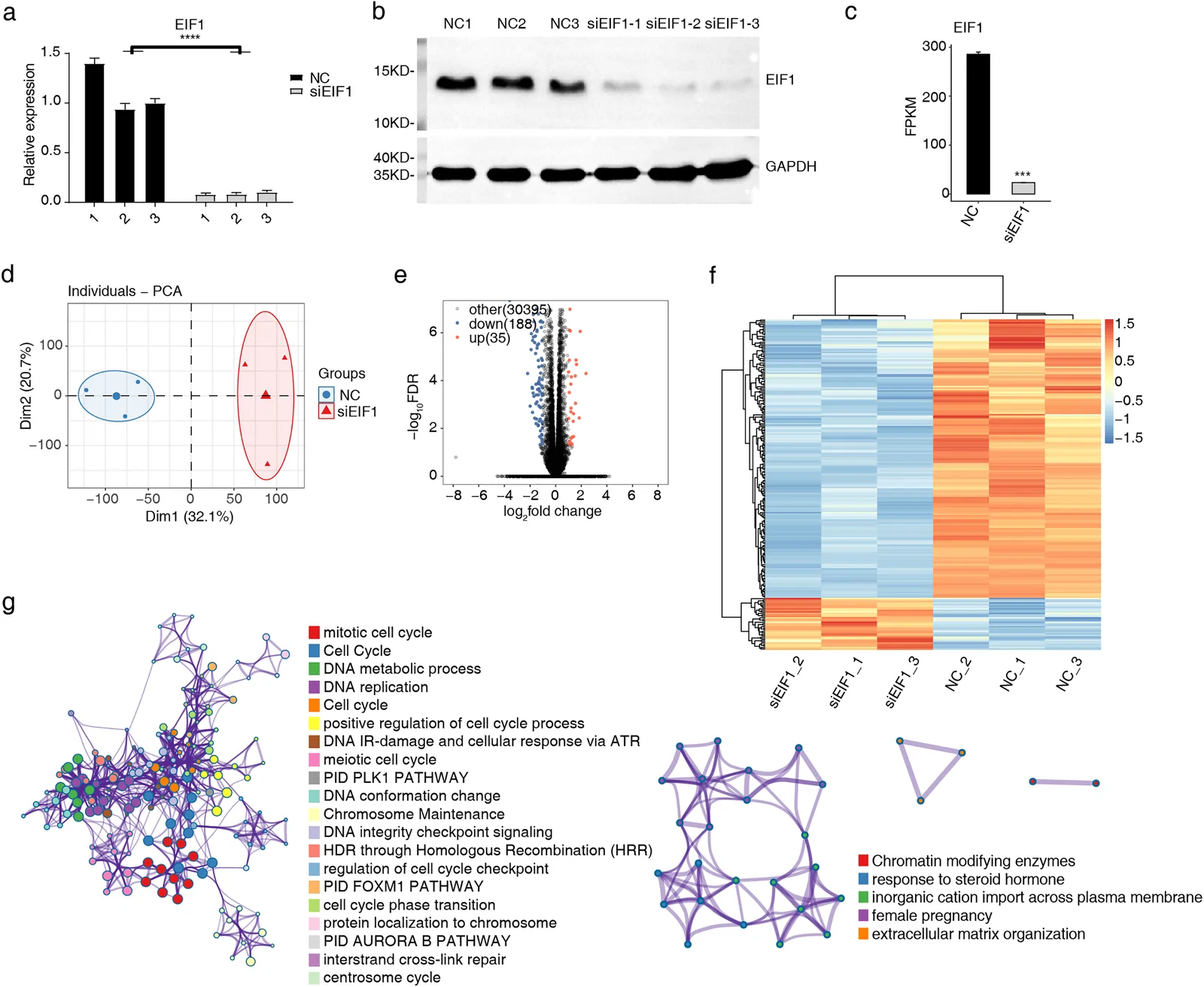
EIF1 regulates gene expression in ARPE-19 cells. a. Validation of EIF1 knockdown efficiency by RT-qPCR. Data are shown as mean ± SD (n = 3 per group). **** P-value < 0001. b. Validation of EIF1 knockdown efficiency by western blot. Data are shown as mean ± SD (n = 3 per group). c. Bar plot showing EIF1 expression levels (in FPKM) from RNA-seq data. Data are shown as mean ± SEM (n = 3 per group). ***P-value < 0.001. d. Principal component analysis (PCA) of samples after normalizing all genes expression levels. The ellipse for each group is the confidence ellipse. e. Volcano plot of DEGs between si-EIF1 and NC groups. Red indicates genes with up regulated expression. Blue indicates genes with down regulated expression. f. Hierarchical clustering heatmap showing expression levels of all DEGs. FPKM values are log2-transformed and then median-centred by each gene. g. Metascape enrichment analysis of upregulated (right) and downregulated (left) DEGs.

To investigate the transcriptional regulatory mechanism of EIF1 in DR, we established a high-glucose (50 mM)-induced ARPE-19 cell injury model and successfully constructed an EIF1-specific knockdown system. Using lipid-mediated siRNA transfection with targeted sequences (siEIF1), both qPCR and western blotting confirmed that the mRNA and protein levels of EIF1 were significantly reduced in the siEIF1 group compared to the negative control (NC) group, validating efficient gene silencing (Fig 2a, 2b).

For systematic transcriptomic profiling, six RNA-seq libraries were constructed from three biological replicates per group. Following FPKM normalization, transcriptomic analysis revealed significant downregulation of EIF1 expression in the siEIF1 group, consistent with qPCR results (Fig 2c). Principal component analysis (PCA) demonstrated distinct clustering of gene expression profiles between the EIF1 knockdown and control groups, indicating global transcriptional remodeling upon EIF1 depletion (Fig 2d). Differential expression analysis using the DESeq2 algorithm (criteria: |FC| ≥ 2 and corrected Pvalue < 0.05) identified 223 significantly dysregulated genes (DEGs), including 35 upregulated and 188 downregulated genes (Fig 2e). Unsupervised hierarchical clustering heatmaps visualized group-specific expression patterns of DEGs (Fig 2f), suggesting that EIF1 knockdown predominantly suppresses gene expression.

**Fig 3:**
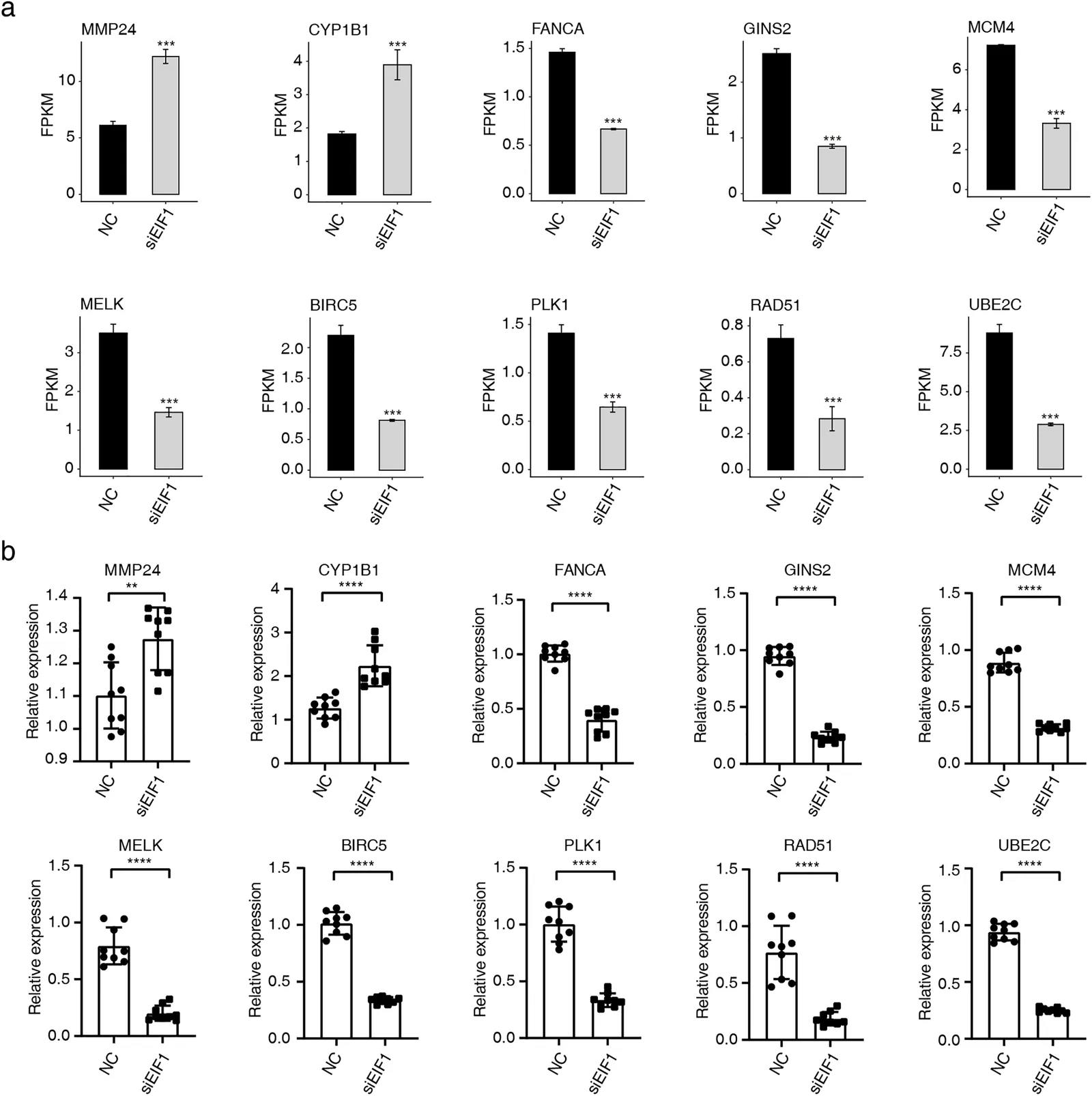
The EIF1 regulates gene expression in ARPE-19 cells. a. Bar plots showing the expression pattern and statistical difference of DEGs. Data are shown as mean ± SEM (n = 3 per group). ***P-value < 0.001. b. RT-qPCR validation of the selected DEGs. Data are shown as mean ± SD (n = 3 per group). ** p < 0.01, **** p < 0.0001.

Functional enrichment analysis of DEGs via Metascape revealed that upregulated genes were significantly enriched in pathways such as chromatin-modifying enzyme activity regulation, steroid hormone response, and inorganic cation transmembrane transport (Fig 2g, right). Downregulated genes were predominantly associated with core cell cycle-related pathways, including mitotic cell cycle, cell cycle, DNA replication, meiotic cell cycle and HDR through homologous recombination repair (HRR) (Fig 2g). Gene Ontology (GO) and Kyoto Encyclopedia of Genes and Genomes (KEGG) analyses further confirmed that downregulated genes were enriched in biological processes such as mitotic spindle organization, chromosome segregation, cell cycle and DNA replication, indicating that EIF1 deficiency markedly inhibits cell cycle-related gene expression (S1 Fig). Further screening revealed that MMP24, CYP1B1 and multiple key genes regulating the cell cycle (including FANCA, GINS2, MCM4, MELK, BIRC5, PLK1, RAD51, UBE2C) showed significantly differential expression after EIF1 knockdown (Fig 3a). To experimentally validate these findings, we performed RT-qPCR on independent biological replicates for the DEGs identified above. The results confirmed differential expression of all 10 genes (FANCA, GINS2, MCM4, MELK, BIRC5, PLK1, RAD51, UBE2C, MMP24, and CYP1B1) in si-EIF1-treated cells compared to controls (p < 0.05 for all), consistent with the RNA-seq data (Fig 3b). These findings suggest that EIF1 may protect retinal pigment epithelial cells from hyperglycemic damage by maintaining the expression homeostasis of cell cycle-related genes.

### 3.3 EIF1 regulates alternative splicing events in ARPE-19 cells

**Fig 4:**
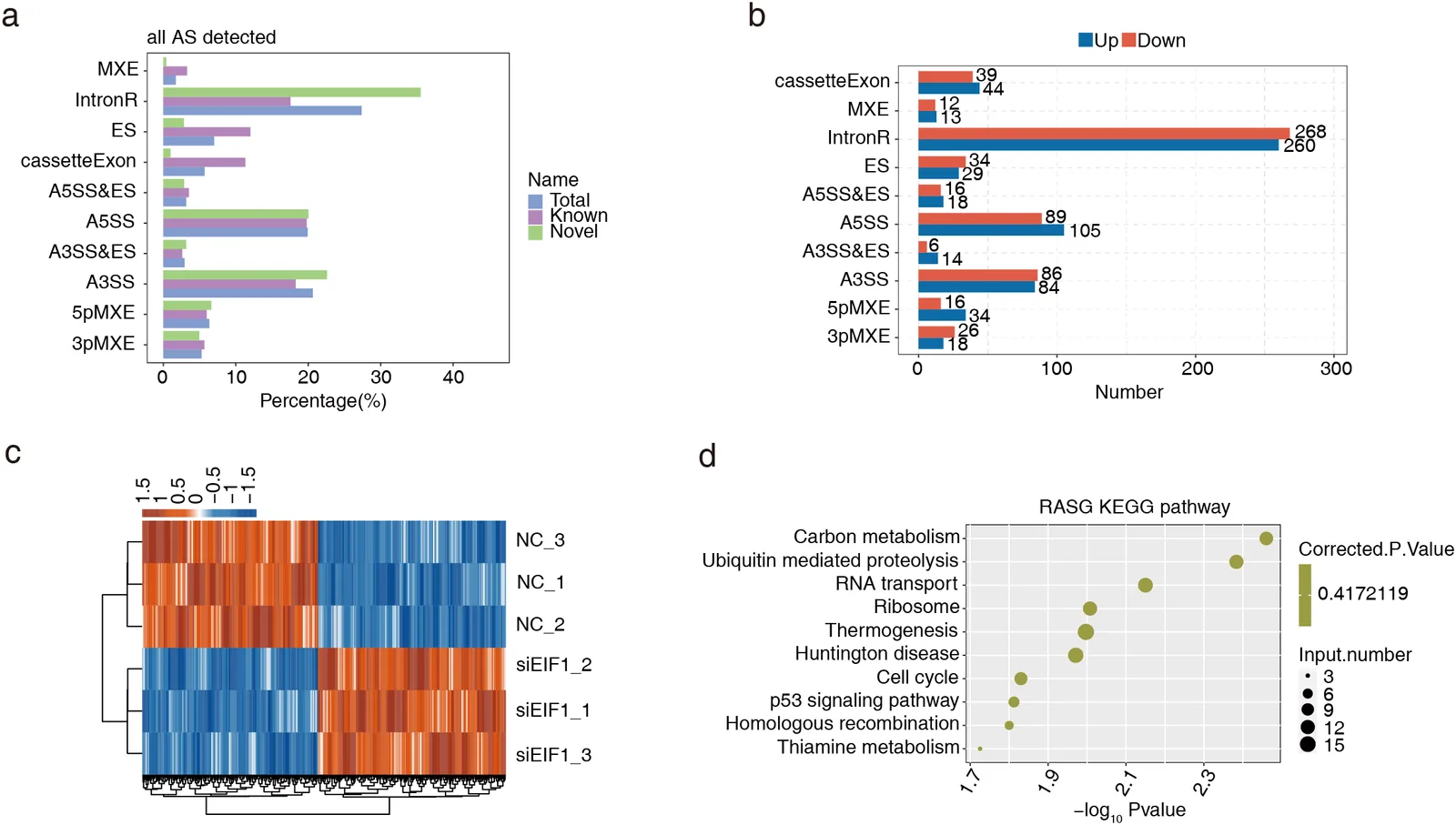
EIF1 regulates gene alternative splicing in ARPE-19 cells. a. Bar plot showing the distribution of all detected ASEs. b. Bar plot showing the distribution of RASEs. c. Hierarchical clustering heatmap of RASEs based on ratio values. Splicing ratio are log2-transformed and then median-centred by each gene. d. Scatter plot exhibiting the most enriched KEGG pathway results of the RASGs.

Previous studies have demonstrated that EIF1 regulates and interacts with AS processes [10]. Leveraging RNA-seq data, we conducted comparative splicing analysis between EIF1-knockdown and control groups, revealing a substantial number of differentially regulated ASEs (Fig 4a). A total of 1211 ASEs exhibited significant alterations in splicing patterns (significance threshold: P ≤ 0.05), comprising 619 upregulated and 592 downregulated events (Fig 4b). The predominant RASE types included IntronR, A5SS, and A3SS (Fig 4b). Hierarchical clustering of these RASEs demonstrated pronounced divergence in splicing profiles between the siEIF1 and NC groups (Fig 4c).

**Fig 5:**
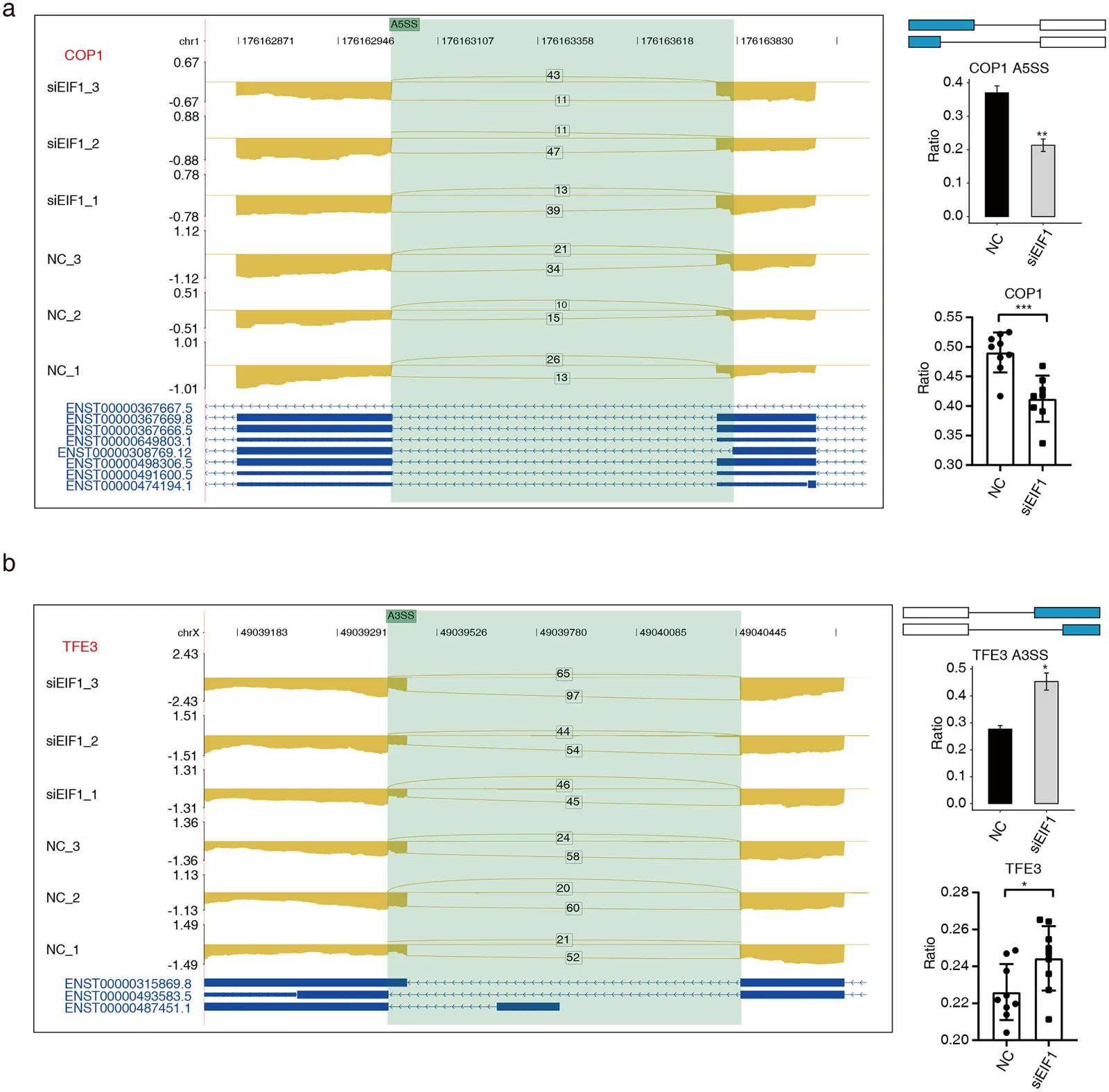
The EIF1 regulates alternative splicing of COP1 and TFE3. a. The EIF1 regulates alternative splicing of COP1. Left panel: IGV-sashimi plot showing the regulated alternative splicing events and binding sites across mRNA. Reads distribution of RASE is plotted in the up panel and the transcripts of each gene are shown below. Right panel: The schematic diagrams depict the structures of ASEs. RNA-seq validation of ASEs are shown at the bottom of the right panel. Error bars represent mean ± SEM. RT-qPCR validation of COP1. Data are shown as mean ± SD (n = 3 per group). ** p < 0.01, *** p < 0.001. b. The EIF1 regulates alternative splicing of TFE3. Left panel: IGV-sashimi plot showing the regulated alternative splicing events and binding sites across mRNA. Reads distribution of RASE is plotted in the up panel and the transcripts of each gene are shown below. Right panel: The schematic diagrams depict the structures of ASEs. RNA-seq validation of ASEs are shown at the bottom of the right panel. Error bars represent mean ± SEM. RT-qPCR validation of TFE3. Data are shown as mean ± SD (n = 3 per group). * p < 0.05.

We then analyzed the regulated alternative splicing genes (RASGs) with significant changes after EIF1 knockdown. KEGG enrichment analysis showed enrichment in cell cycle and p53 signaling pathway (Fig 4d). GO enrichment analysis showed enrichment in DNA repair, cellular response to DNA damage stimulus and DNA replication (S2a Fig). Metascape enrichment analysis showed that DNA metabolic process, Metabolism of RNA were the main biological processes involved (S2b Fig). Based on literature research and screening of supporting reads, read ratios (ratio values), and their changes before and after EIF1 knockdown, we finally selected 5 AS events and their associated genes: MCM7, COP1, CPSF4, TFE3, and HMGA1. RNA-seq read distribution confirmed robust splicing isoform changes in these genes, which are implicated in diabetes and related metabolic disorders (Fig 5a, 5b, S3a–S3c Fig). To experimentally validate these splicing events, we performed RT-qPCR with isoform-specific primers on independent biological replicates. The results confirmed significant alterations in splicing patterns for COP1 and TFE3 (p < 0.05; Fig 5a, 5b), consistent with the RNA-seq predictions. Unfortunately, the remaining three candidate AS events (MCM7, CPSF4, and HMGA1) did not show consistent splicing changes by RT-qPCR. This discrepancy may be attributed to the inherent limitations of computational prediction from short-read RNA-seq data, which can yield false-positive AS events, or to relatively low abundance of the spliced isoforms that falls below the reliable detection threshold of qPCR. These unconfirmed events are presented in Supplementary S3a–S3c Fig for completeness. Collectively, the observed alternative splicing alterations in these genes exhibit significant associations with cell cycle, potentially implicating their dysregulation in the pathogenesis of hyperglycemia-induced retinal degeneration.

## 4 Discussion

DR, one of the most severe microvascular complications of diabetes mellitus, remains incompletely understood in terms of its precise pathological mechanisms [12]. It is now well-established that DR is a multifactorial disease involving the complex interplay of various retinal cell types, including vascular endothelial cells, pericytes, neurons, and glial cells, with pathways of inflammation, oxidative stress, and angiogenesis playing central roles [13,14]. While vascular dysfunction has historically been considered the primary driver of DR, accumulating evidence highlights the critical contribution of the retinal pigment epithelium to retinal health and its susceptibility to diabetic insult [15]. Eukaryotic translation initiation factor EIF1, a core regulator of protein synthesis, not only participates in fundamental translational activities by modulating ribosome recruitment and mRNA scanning processes but has also recently been implicated in critical pathological pathways such as cell cycle regulation and DNA damage repair [16,17]. Notably, Zhao et al. first identified a significant inverse correlation between EIF1 expression levels and glucotoxic conditions through transcriptomic analysis of high glucose-stimulated human retinal endothelial cells (hRECs) [10], suggesting EIF1 as a potential therapeutic target for DR intervention. However, its specific regulatory network requires further elucidation.

In this study, we established a high-glucose-cultured ARPE-19 cell model to investigate the functional role of EIF1 in DR. Transcriptomic profiling following EIF1 knockdown revealed a predominant downregulation of gene expression. Functional enrichment analysis demonstrated that these DEGs were strongly associated with cell cycle regulation, a pathway critically implicated in DR pathogenesis [18]. Existing literature underscores that dysregulation of the retinal epithelial cell cycle constitutes a core pathological mechanism in DR. Under hyperglycemic stress, the CDK4/6-Cyclin D1 signaling axis is aberrantly activated, driving defective G1/S phase transition in retinal pigment epithelial cells and resulting in uncontrolled proliferation [19–21]. Our findings align with these observations, suggesting that EIF1 may exert a unique regulatory role in DR by modulating cell cycle-related genes. Specifically, the coordinated downregulation of EIF1-dependent targets under high-glucose conditions likely disrupts cell cycle checkpoints, exacerbating retinal epithelial dysfunction. These results suggest that EIF1 may be associated with cell cycle dysregulation in DR, offering novel insights for therapeutic targeting of cell cycle machinery in diabetic microvascular complications.

Further analysis of differentially expressed genes following EIF1 knockdown revealed significant transcriptional alterations in key cell cycle regulators, including BIRC5, MCM4, PLK1, RAD51, MELK, UBE2C, GINS2, and FANCA. Notably, MCM4 and GINS2 are both components of the replicative helicase complex required for DNA replication initiation; their downregulation has been observed in hyperglycemic or diabetic models and is associated with cell cycle arrest and attenuated proliferative capacity [22,23]. PLK1 and MELK are mitotic kinases that regulate complementary steps of M-phase progression; reduced PLK1 expression in retinal pigment epithelial cells may exacerbate hyperglycemia-induced cellular damage, while MELK downregulation similarly impairs mitotic progression [24–26]. RAD51 and FANCA function in homologous recombination repair pathways; RAD51 depletion disrupts DNA repair and accelerates apoptosis under hyperglycemic stress [27,28], and FANCA knockdown compromises DNA repair capacity in the context of type 2 diabetes [29]. BIRC5 coordinates apoptosis inhibition and chromosome segregation during mitosis; its downregulation correlates with elevated apoptotic susceptibility in diabetic conditions [30]. All of these DEGs were successfully validated by RT‑qPCR, confirming the robustness of our transcriptomic data. Collectively, these findings suggest that EIF1 is associated with ARPE-19 cell cycle progression and proliferative capacity through associations with the transcriptional regulation of these effectors, potentially influencing the pathophysiological trajectory of DR.

AS analysis revealed that EIF1 knockdown significantly modulates a spectrum of differential splicing events. Functional annotation of these alternatively spliced genes demonstrated robust enrichment in DNA damage response and cell cycle regulation, highlighting EIF1’s dual roles in maintaining genomic integrity and proliferative homeostasis. Notably, our alternative splicing analysis, followed by experimental validation, identified COP1 and TFE3 as targets associated with EIF1-mediated splicing regulation. COP1 is an E3 ubiquitin ligase that has been shown to regulate cell cycle progression through its interaction with key regulators such as p53 [31]. TFE3 is a transcription factor that regulate cell cycle progression through the transcriptional activation of p21 [32]. Based on these observations, we hypothesize that EIF1-mediated alternative splicing of COP1 and TFE3 may generate functionally distinct protein isoforms that contribute to cell cycle dysregulation in DR. Further functional studies, including overexpression and rescue experiments, are needed to clarify the specific roles of these splicing variants in the context of DR.

Although our findings suggest potential relevance for understanding DR pathogenesis, several limitations warrant consideration. First, our study was conducted exclusively in ARPE-19 cells, a transformed cell line that may not fully replicate the behavior of primary RPE cells or the complex in vivo environment of the diabetic retina. Therefore, our findings should be considered as hypothesis-generating and require validation in primary RPE cultures and in vivo animal models of DR. Second, the use of 50 mM glucose without an equimolar osmotic control (e.g., mannitol) is a significant confound. We cannot exclude the possibility that some of the observed effects on cell proliferation, EIF1 expression, and the transcriptome may be partly attributable to hyperosmotic stress rather than the specific metabolic effects of high glucose. Future studies must include appropriate osmotic controls to dissect these distinct pathways. Third, our data demonstrate association rather than causation, and we cannot yet distinguish whether EIF1 regulates downstream gene expression or splicing through direct RNA binding or indirectly via downstream effectors.. Future studies CLIP-seq or iRIP-seq to map EIF1’s direct RNA targets, along with cell cycle profiling, rescue experiments, and perturbation of downstream candidates, will be required to establish both the direct-versus-indirect mechanisms and the causal role of EIF1 in DR pathogenesis. Finally, the translational relevance of EIF1 as a therapeutic target remains speculative without confirmation in human samples or animal models. Nevertheless, this study is the first to associate EIF1 with cell cycle regulation in retinal pigment epithelial cells under hyperglycemic conditions, providing candidate molecules and transcriptomic data that may guide subsequent exploration of novel therapeutic targets for DR.

## 5 Conclusion

In summary, this study suggests that EIF1 is downregulated under high-glucose conditions and may regulate the expression and alternative splicing of cell cycle-related genes in ARPE-19 cells. These findings point to EIF1 as a candidate worthy of further investigation in DR pathogenesis.

**Key messages**

**What is known**

- Diabetic retinopathy is one of the most severe microvascular complications of diabetes mellitus. EIF1 is an RNA-binding protein that has been found to be involved in a variety of diseases, but its specific role in diabetic retinopathy remains unclear.

**What is new**

- In a high glucose-induced retinal injury cell model, EIF1 expression was significantly inhibited accompanied by reduced cell proliferation, suggesting that hyperglycemic conditions may promote EIF1 degradation, thereby impairing retinal pigment epithelial cell function.
- Through high-throughput sequencing technology, we found that EIF1 mediates hyperglycemia-induced dysfunction of retinal pigment epithelial cells by mediating the differential expression and alternative splicing of cell cycle-related genes.

## Supporting information

S1 Fig Functional enrichment analysis of DEGs upon EIF1 knockdown. a. The Scatter plot exhibiting the most enriched GO pathway results of the down-regulated DEGs. b. The Scatter plot exhibiting the most enriched KEGG pathway results of the DEGs.
(TIF)

S2 Fig Functional enrichment analysis reveals key pathways modulated by RASGs upon EIF1 knockdown. a. Scatter plot exhibiting the most enriched GO biological process results of the RASGs. b. Metascape enrichment analysis map showing the RASGs.
(TIF)

S3 Fig The EIF1 regulates alternative splicing of MCM7, CPSF4 and HMGA1. a. The EIF1 regulates alternative splicing of MCM7. Left panel: IGV-sashimi plot showing the regulated alternative splicing events and binding sites across mRNA. Reads distribution of RASE is plotted in the up panel and the transcripts of each gene are shown below. Right panel: The schematic diagrams depict the structures of ASEs. RNA-seq validation of ASEs are shown at the bottom of the right panel. Error bars represent mean ± SEM. RT-qPCR validation of MCM7. Data are shown as mean ± SD (n = 3 per group). ** p < 0.01, *** p < 0.001. b. The EIF1 regulates alternative splicing of CPSF4. Left panel: IGV-sashimi plot showing the regulated alternative splicing events and binding sites across mRNA. Reads distribution of RASE is plotted in the up panel and the transcripts of each gene are shown below. Right panel: The schematic diagrams depict the structures of ASEs. RNA-seq validation of ASEs are shown at the bottom of the right panel. Error bars represent mean ± SEM. RT-qPCR validation of CPSF4. Data are shown as mean ± SD (n = 3 per group). * p < 0.05. c. The EIF1 regulates alternative splicing of HMGA1. Left panel: IGV-sashimi plot showing the regulated alternative splicing events and binding sites across mRNA. Reads distribution of RASE is plotted in the up panel and the transcripts of each gene are shown below. Right panel: The schematic diagrams depict the structures of ASEs. RNA-seq validation of ASEs are shown at the bottom of the right panel. Error bars represent mean ± SEM. RT-qPCR validation of HMGA1. Data are shown as mean ± SD (n = 3 per group). * p < 0.05.
(TIF)

S1 Table The sequence table of primers used in this study for RT-qPCR experiment. (DOCX)

S1 Raw images The original image of Western blot of Fig 2B before clipping. (PDF)
