# Supplementary material for: EIF1 coordinates transcriptomic and splicing networks associated with cell cycle dysregulation in diabetic retinopathy
Source: PLoS One. 2026 Sep 3;21(9):e0355473. doi: 10.1371/journal.pone.0355473 (PMC13541169; doi:10.1371/journal.pone.0355473)

The original image of Western blot of Figure 2B before clipping.

Groups:

1.si EIF1 1-1

2. si EIF1 1-2

3. si EIF1 1-3

4. NC-1

5. NC-2

6. NC-3

7. si EIF1 2-1

8. si EIF1 2-2

9. si EIF1 2-3

EIF1

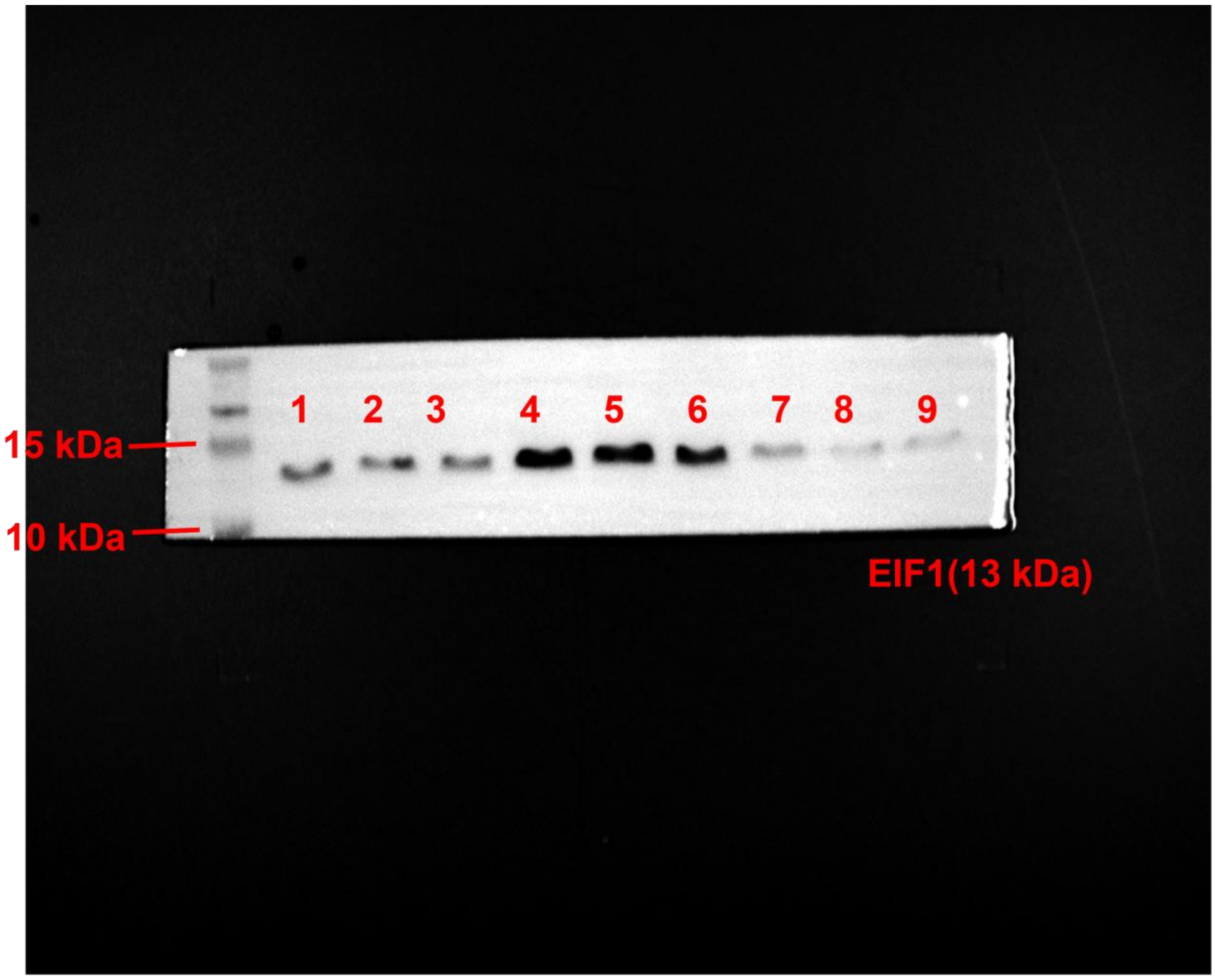

GAPDH

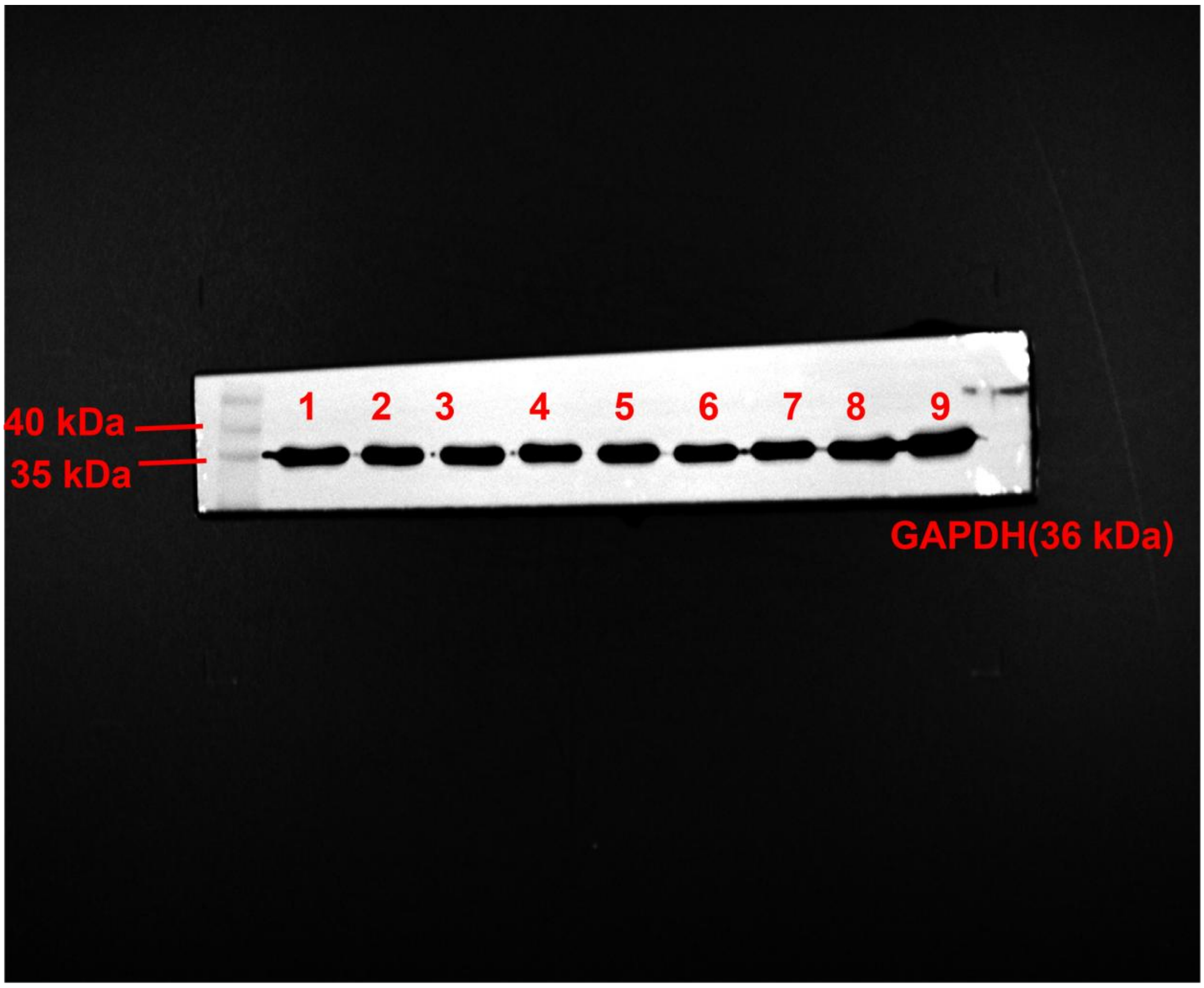

Supplement: Supplementary file 1 [file pone.0355473.s005.pdf]
