# Supplementary material for: EIF1 coordinates transcriptomic and splicing networks associated with cell cycle dysregulation in diabetic retinopathy
Source: PLoS One. 2026 Sep 3;21(9):e0355473. doi: 10.1371/journal.pone.0355473 (PMC13541169; doi:10.1371/journal.pone.0355473)
Supplement: Supplementary file 2 [file pone.0355473.s004.docx]

**Table S1. The sequence table of primers used in this study for RT-qPCR experiment.**

| Primers | Sequence | Length (bp) |
| --- | --- | --- |
| GAPDH-F | GGTCGGAGTCAACGGATTTG | 218 |
| GAPDH-R | GGAAGATGGTGATGGGATTTC |  |
| EIF1-F | GGTACTGTAATTGAGCATCC | 101 |
| EIF1-R | TTAGCCAGTCCAATCTCTAC |  |
| CYP1B1-F: | TTTGGTAATGGTGTCCCAGTAT | 143 |
| CYP1B1-R: | GTGTGTGCATAATAGCTACAGT |  |
| MCM4-F: | TGGATTTGATGACAGGCATAGC | 140 |
| MCM4-R: | AGCCGTGATTGTACCAGTGT |  |
| MELK-F: | TATCAGGAGGCAGCGGCTTA | 127 |
| MELK-R: | TGTATCACACCCACACTCATCC |  |
| RAD51-F: | GCTGAAGCTATGTTCGCCATT | 155 |
| RAD51-R: | GCAGTCACAACAGGAAGAGG |  |
| PLK1-F: | TCCAGAAGATGCTTCAGACAGA | 121 |
| PLK1-R: | GGAATGGTCAGGCAGGTGAT |  |
| GINS2-F: | GCTCTGCTAGGGAGAATGACT | 129 |
| GINS2-R: | GGATTCAGGATGTTGTTGGAGA |  |
| FANCA-F: | AGAGGTGCGGAACTATATCTGT | 121 |
| FANCA-R: | GGTCCTGACTCACACTTACTG |  |
| BIRC5-F: | GCACAAAGCCATTCTAAGTCAT | 121 |
| BIRC5-R: | TGTCTAATCACACAGCAGTGG |  |
| MMP24-F: | CCTAGAGGCTTCCAATCTCAGA | 155 |
| MMP24-R: | ATGTGACTGACACTGAAATGCT |  |
| UBE2C-F: | CGCCCACAGTGAAGTTCCT | 131 |
| UBE2C-R: | CTGGATGGAGAGCAGAATGGT |  |
| COP1-M/AS-F: | CTTCCAGTTGTTTCTTCTTC | M: 129  AS: 130 |
| COP1-M-R: | TGAGTAGCACCAATGGCCACA |  |
| COP1-AS-R: | TTGGACCACTCAAATGGCCACA |  |
| TFE3-AS-F: | CTGGAGCCCCCTTGAGCGAA | M: 90  AS: 90 |
| TFE3-M-F: | TATTGGGAGGCTTGAGCGAA |  |
| TFE3-M/AS-R: | GCTGACATAGAATTAGAAAACG |  |
| MCM7-M/AS-F: | TGCTTCTTCCCGAGTTCATC | M: 68  AS: 68 |
| MCM7-AS-R: | GGAGACTTCGAAAAGGTTAA |  |
| MCM7-M-R: | CTAGAGAAGGAAAAGGTTAA |  |
| CPSF4-M/AS-F: | CATGGGAACCACCGAGCAGC | M: 66  AS: 66 |
| CPSF4-M-R: | TGTTACTTTGCTTTGCTGGA |  |
| CPSF4-AS-R: | TGCGGGGTTCTCTTTGCTGGA |  |
| HMGA1-M-F: | CTTATATGAGCATCCCAGCCA | M: 124  AS: 125 |
| HMGA1-AS-F: | TGGGATTACAGCATCCCAGCCA |  |
| HMGA1-M/AS-R: | GCTTCTCAGTGCCGTCCTTTT |  |

AS, alternative splicing; F, forward prime; R, reverse primer; M, model
