# Supplementary material for: EIF1 coordinates transcriptomic and splicing networks associated with cell cycle dysregulation in diabetic retinopathy
Source: PLoS One. 2026 Sep 3;21(9):e0355473. doi: 10.1371/journal.pone.0355473 (PMC13541169; doi:10.1371/journal.pone.0355473)
Supplement: Supplementary file 3 [file pone.0355473.s006.docx]

PONE-D-25-49013

EIF1 coordinates transcriptomic and splicing networks to drive cell cycle dysregulation in diabetic retinopathy

PLOS One

Dear Dr. Li,

Thank you for submitting your manuscript to PLOS ONE. After careful consideration, we feel that it has merit but does not fully meet PLOS ONE’s publication criteria as it currently stands. Therefore, we invite you to submit a revised version of the manuscript that addresses the points raised during the review process.

Please submit your revised manuscript by Mar 16 2026 11:59PM. If you will need more time than this to complete your revisions, please reply to this message or contact the journal office at [plosone@plos.org](mailto:plosone@plos.org). When you're ready to submit your revision, log on to https://www.editorialmanager.com/pone/ and select the 'Submissions Needing Revision' folder to locate your manuscript file.

Please include the following items when submitting your revised manuscript:

- A letter that responds to each point raised by the academic editor and reviewer(s). You should upload this letter as a separate file labeled 'Response to Reviewers'.
- A marked-up copy of your manuscript that highlights changes made to the original version. You should upload this as a separate file labeled 'Revised Manuscript with Track Changes'.
- An unmarked version of your revised paper without tracked changes. You should upload this as a separate file labeled 'Manuscript'.

If you would like to make changes to your financial disclosure, please include your updated statement in your cover letter. Guidelines for resubmitting your figure files are available below the reviewer comments at the end of this letter.

If applicable, we recommend that you deposit your laboratory protocols in protocols.io to enhance the reproducibility of your results. Protocols.io assigns your protocol its own identifier (DOI) so that it can be cited independently in the future. For instructions see: [https://journals.plos.org/plosone/s/submission-guidelines#loc-laboratory-protocols](https://journals.plos.org/plosone/s/submission-guidelines#loc-laboratory-protocols). Additionally, PLOS ONE offers an option for publishing peer-reviewed Lab Protocol articles, which describe protocols hosted on protocols.io. Read more information on sharing protocols at <https://plos.org/protocols?utm_medium=editorial-email&utm_source=authorletters&utm_campaign=protocols>.

We look forward to receiving your revised manuscript.

Kind regards,

Kangkang Ji, Ph.D., M.D.
Academic Editor
PLOS One

**Response:**

Dear Dr. Ji and Reviewers,

We sincerely thank you for the valuable time and insightful comments on our manuscript. We have carefully considered all the suggestions and have revised the manuscript accordingly. Below is a point-by-point response to each comment raised by the reviewers and the editorial office. All changes in the manuscript are highlighted in blue font for easy identification.

**Response to Editorial Office Requirements**

Journal Requirements:

When submitting your revision, we need you to address these additional requirements.

1. Please ensure that your manuscript meets PLOS ONE's style requirements, including those for file naming. The PLOS ONE style templates can be found at https://journals.plos.org/plosone/s/file?id=wjVg/PLOSOne_formatting_sample_main_body.pdf and https://journals.plos.org/plosone/s/file?id=ba62/PLOSOne_formatting_sample_title_authors_affiliations.pdf

**Response:** We have reviewed the PLOS ONE formatting templates and ensured that our manuscript meets the style requirements, including file naming and structure.

2. Please note that PLOS One has specific guidelines on code sharing for submissions in which author-generated code underpins the findings in the manuscript. In these cases, we expect all author-generated code to be made available without restrictions upon publication of the work. Please review our guidelines at https://journals.plos.org/plosone/s/materials-and-software-sharing#loc-sharing-code and ensure that your code is shared in a way that follows best practice and facilitates reproducibility and reuse.

**Response:** Our study did not generate custom code; all analyses were performed using publicly available tools as described in the Methods section. No code sharing is applicable.

3. Please update your submission to use the PLOS LaTeX template. The template and more information on our requirements for LaTeX submissions can be found at <http://journals.plos.org/plosone/s/latex.>

**Response:** Thank you for reminding us about the PLOS LaTeX template. We have carefully reviewed the guidelines at the provided link.

We would like to inform the editor that our revised manuscript has been prepared using Microsoft Word and has been carefully formatted to meet all PLOS ONE style requirements as outlined in the journal's formatting templates.

Given that our manuscript is fully prepared in Word and contains no complex mathematical or scientific notation that would necessitate LaTeX, we respectfully request permission to submit the manuscript in Word format. The manuscript file, figures, and supporting information are ready for submission as per the standard procedure. We believe this format will not compromise the review or production process. If the editor insists on a LaTeX submission, we will of course comply and undertake the necessary conversion.

Thank you for your understanding.

4. Thank you for stating the following in the Acknowledgments Section of your manuscript:
“This study was supported by the Natural Science Foundation of Ningxia (Grant No. [2022AAC03564])”
We note that you have provided funding information that is not currently declared in your Funding Statement. However, funding information should not appear in the Acknowledgments section or other areas of your manuscript. We will only publish funding information present in the Funding Statement section of the online submission form.
Please remove any funding-related text from the manuscript and let us know how you would like to update your Funding Statement. Currently, your Funding Statement reads as follows:
“This study was supported by the Natural Science Foundation of Ningxia (Grant No. [2022AAC03564]).”
Please include your amended statements within your cover letter; we will change the online submission form on your behalf.

**Response:**

We would like to respectfully clarify that in our submitted manuscript, the Acknowledgments section actually reads "None." , and the funding information was placed in a separate "Funding" section after the Acknowledgments, as is common practice in many journals.

Nevertheless, we understand and comply with PLOS ONE's policy that funding information should not appear in the manuscript text but should only be declared in the online submission system. Therefore, we have:

1. Removed the entire "Funding" section from the manuscript
2. Included the funding statement in our cover letter as requested
3. Confirmed that the online submission form will be updated accordingly with the following statement:

"This study was supported by the Natural Science Foundation of Ningxia (Grant No. [2022AAC03564]). The funders had no role in study design, data collection and analysis, decision to publish, or preparation of the manuscript."

We appreciate the editor's attention to this detail and have made the necessary corrections.

5. We note that the grant information you provided in the ‘Funding Information’ and ‘Financial Disclosure’ sections do not match.
When you resubmit, please ensure that you provide the correct grant numbers for the awards you received for your study in the ‘Funding Information’ section.

**Response:** We have verified that the grant information in the ‘Funding Information’ and ‘Financial Disclosure’ sections match. The correct grant number is [2022AAC03564].

6. Thank you for stating the following financial disclosure:
“This study was supported by the Natural Science Foundation of Ningxia (Grant No. [2022AAC03564]).”
Please state what role the funders took in the study.  If the funders had no role, please state: "The funders had no role in study design, data collection and analysis, decision to publish, or preparation of the manuscript."
If this statement is not correct you must amend it as needed.
Please include this amended Role of Funder statement in your cover letter; we will change the online submission form on your behalf.

**Response:** As stated above, we have included the role statement in the cover letter. The funders had no role in the study.

7. PLOS ONE now requires that authors provide the original uncropped and unadjusted images underlying all blot or gel results reported in a submission’s figures or Supporting Information files. This policy and the journal’s other requirements for blot/gel reporting and figure preparation are described in detail at https://journals.plos.org/plosone/s/figures#loc-blot-and-gel-reporting-requirements and https://journals.plos.org/plosone/s/figures#loc-preparing-figures-from-image-files. When you submit your revised manuscript, please ensure that your figures adhere fully to these guidelines and provide the original underlying images for all blot or gel data reported in your submission. See the following link for instructions on providing the original image data: https://journals.plos.org/plosone/s/figures#loc-original-images-for-blots-and-gels.
In your cover letter, please note whether your blot/gel image data are in Supporting Information or posted at a public data repository, provide the repository URL if relevant, and provide specific details as to which raw blot/gel images, if any, are not available. Email us at plosone@plos.org if you have any questions.

**Response:** All original uncropped and unadjusted images for western blots are provided in the Supporting Information file.

8. PLOS requires an ORCID iD for the corresponding author in Editorial Manager on papers submitted after December 6th, 2016. Please ensure that you have an ORCID iD and that it is validated in Editorial Manager. To do this, go to ‘Update my Information’ (in the upper left-hand corner of the main menu), and click on the Fetch/Validate link next to the ORCID field. This will take you to the ORCID site and allow you to create a new iD or authenticate a pre-existing iD in Editorial Manager.

**Response:** The corresponding author (Na Li) has validated her ORCID iD (0009-0007-3141-0124 ) in Editorial Manager.

9. If the reviewer comments include a recommendation to cite specific previously published works, please review and evaluate these publications to determine whether they are relevant and should be cited. There is no requirement to cite these works unless the editor has indicated otherwise.
**Response:** The reviewers did not specifically request citation of any particular published works, so no additional citations have been added.

[Note: HTML markup is below. Please do not edit.]

Reviewers' comments:

Reviewer's Responses to Questions

**Comments to the Author**

1. Is the manuscript technically sound, and do the data support the conclusions?

The manuscript must describe a technically sound piece of scientific research with data that supports the conclusions. Experiments must have been conducted rigorously, with appropriate controls, replication, and sample sizes. The conclusions must be drawn appropriately based on the data presented.

Reviewer #1: Yes

Reviewer #2: Yes

2. Has the statistical analysis been performed appropriately and rigorously?

Reviewer #1: I Don't Know

Reviewer #2: Yes

3. Have the authors made all data underlying the findings in their manuscript fully available?

The [PLOS Data policy](http://www.plosone.org/static/policies.action#sharing) requires authors to make all data underlying the findings described in their manuscript fully available without restriction, with rare exception (please refer to the Data Availability Statement in the manuscript PDF file). The data should be provided as part of the manuscript or its supporting information, or deposited to a public repository. For example, in addition to summary statistics, the data points behind means, medians and variance measures should be available. If there are restrictions on publicly sharing data—e.g. participant privacy or use of data from a third party—those must be specified.

Reviewer #1: Yes

Reviewer #2: Yes

4. Is the manuscript presented in an intelligible fashion and written in standard English?

PLOS ONE does not copyedit accepted manuscripts, so the language in submitted articles must be clear, correct, and unambiguous. Any typographical or grammatical errors should be corrected at revision, so please note any specific errors here.

Reviewer #1: Yes

Reviewer #2: Yes

5. Review Comments to the Author

Please use the space provided to explain your answers to the questions above. You may also include additional comments for the author, including concerns about dual publication, research ethics, or publication ethics. (Please upload your review as an attachment if it exceeds 20,000 characters)

Reviewer #1: Overall this is a well-written paper that presents some important results. There are a few mostly minor things:
1) Western in western blotting should not be capitalized. Southern in Southern blotting is capitalized since the technique was invented by Dr. Southern. Northern blotting and western blotting were so named as a kind of scientific joke (not invented by a Dr. Northern and Dr. Western). This is not a rare error in scientific writing.

**Response:** We thank the reviewer for pointing out this common error. We have corrected “Western blot” to “western blot” throughout the manuscript. Page 4, Line 104; Page 9, Line 230; Page 17, Line 510.

2) In the Cell Proliferation assay, I would change the last sentence to say the "The net cell proliferation rate..." rather than "The cell proliferation rate..." since the CCK-8 assay results are proportional to the number of viable cells and therefore cannot distinguish whether there was just a change in in proliferation or if cell death was also involved.

**Response:** We agree with the reviewer’s suggestion. We have revised the description in manuscript to use “net cell proliferation rate” to reflect that CCK-8 measures viable cell number (Page 4, Line 113; Page 8, Line 215), which can be affected by both proliferation and cell death.

3) For evaluating RNA integrity before RNA sequencing, calculating an RNA integrity number would have been preferable to just using the A260/280 ration and (visually?) examining RNA gels.

**Response:** We thank the reviewer for this valuable suggestion. We fully acknowledge that the RNA Integrity Number (RIN) is currently the gold standard for assessing RNA quality, providing a more precise and objective measure of RNA integrity.

In our study, we assessed RNA integrity using A260/A280 ratios combined with agarose gel electrophoresis. The detailed quality control results for all six samples (three si-NC and three si-EIF1 biological replicates) are as follows:

| **Sample** | **Sample Name** | **Concentration（**ng/ul） | **A260/A280** | **Volume（ul)** | **Total Amount（ug)** | **Overall Conclusion** |
| --- | --- | --- | --- | --- | --- | --- |
| 1 | ARPE-19 si-NC-1 | 174.24 | 1.995 | 9.5 | 1.66 | Low risk |
| 2 | ARPE-19 si-NC-2 | 225.20 | 2.112 | 9.5 | 2.14 | Qualified |
| 3 | ARPE-19 si-NC-3 | 231.80 | 2.056 | 9.5 | 2.20 | Qualified |
| 4 | ARPE-19 si-EIF1-3-1 | 209.28 | 2.141 | 9.5 | 1.99 | Low risk |
| 5 | ARPE-19 si-EIF1-3-2 | 227.12 | 2.008 | 9.5 | 2.16 | Qualified |
| 6 | ARPE-19 si-EIF1-3-3 | 242.40 | 2.085 | 9.5 | 2.30 | Qualified |

As shown above, all samples exhibited A260/A280 ratios between 1.995 and 2.141, indicating high purity with minimal protein or organic solvent contamination. Additionally, agarose gel electrophoresis revealed clear, distinct ribosomal RNA bands (28S and 18S) with no visible degradation or smearing, and the bands were consistently observed in the 300-400 bp range, confirming that the RNA was intact and suitable for downstream library preparation and sequencing.

We agree with the reviewer that RIN provides a more standardized and quantitative measure of RNA integrity. Nevertheless, the combination of high A260/A280 ratios and clear gel electrophoresis results in this study provides sufficient evidence that the RNA quality was adequate for reliable RNA-seq analysis.

We appreciate the reviewer's attention to this important technical detail.

4) In the Statistical analysis section of Materials and methods, did the authors mean to say that the data was presented as the mean plus and minus the mean sample standard deviation or the standard error of the mean? This should be reworded for clarity. I would note that it is generally more appropriate to show the mean plus or minus the standard error of the mean (SEM). I would also indicate sample sizes in the figure legends, where appropriate. Where a 2-way ANOVA was significant, what method was used for subsequent multiple comparisons?

**Response:** We thank the reviewer for this important methodological observation. We have carefully reviewed our data presentation and clarified the statistical reporting throughout the manuscript. Page 8, Line 202-209.

Regarding the use of SD vs. SEM:

For cellular experimental data (e.g., CCK-8 proliferation assays and RT-qPCR validation experiments), data are presented as mean ± standard deviation (SD). This reflects the variability among independent biological replicates and is appropriate for experiments with a limited number of samples (n=3).

For RNA-seq derived expression values (e.g., FPKM bar plots), data are presented as mean ± standard error of the mean (SEM). This is consistent with standard practice in transcriptomic studies to illustrate the precision of the estimated mean from the sequencing data.

We have now explicitly stated this distinction in the Statistical analysis section and figure legands to avoid any ambiguity.

For two-way ANOVA analyses, we used Tukey's multiple comparisons test as the post-hoc method. This is now clearly stated in the revised Methods section.

We appreciate the reviewer's attention to this detail, which has helped us improve the clarity and rigor of our statistical reporting.

Reviewer #2:The study reports that high-glucose treatment suppresses EIF1 expression in ARPE-19 cells and that EIF1 knockdown under hyperglycemic conditions leads to widespread changes in gene expression and alternative splicing, with enrichment in cell cycle-related pathways. The authors propose that EIF1 coordinates transcriptional and splicing networks that contribute to cell cycle dysregulation in diabetic retinopathy and suggest EIF1 as a potential therapeutic target.

I have a few major concerns and suggestions as following.
-The biological model is limited to a single transformed retinal pigment epithelial cell line (ARPE-19). While commonly used, this line is known to differ substantially from primary RPE in cell cycle behavior, metabolic responses, and differentiation status. Claims regarding diabetic retinopathy pathogenesis should therefore be substantially tempered, or additional validation in primary RPE cells or in vivo models should be included.

**Response:** We fully agree with the reviewer that the use of a single transformed cell line is a significant limitation. ARPE-19 cells, while a standard model, cannot fully recapitulate the complexity of diabetic retinopathy (DR) pathogenesis in vivo. We have substantially tempered our claims throughout the manuscript to reflect that our findings are based on this specific in vitro model. We have explicitly stated this limitation in the Discussion and framed our conclusions as hypotheses that require validation. The following changes have been made:

1)In the Abstract: Changed "This study highlights EIF1’s crucial role in DR..." to "This study suggests a potential role for EIF1 in DR pathogenesis in an in vitro model, identifying it as a candidate for further investigation. The data point to an association between EIF1 and the regulation of cell cycle-related genes at both the transcriptional and splicing levels." Page 2, Line 50-53

2)In the Discussion: Added a sentence to the limitations paragraph: "First, our study was conducted exclusively in ARPE-19 cells, a transformed cell line that may not fully replicate the behavior of primary RPE cells or the complex in vivo environment of the diabetic retina. Therefore, our findings should be considered as hypothesis-generating and require validation in primary RPE cultures and in vivo animal models of DR." Page 14, Line 368-372

-The use of 50 mM glucose as the primary experimental condition raises concerns about osmotic stress rather than physiological hyperglycemia. The absence of an osmotic control (e.g., mannitol) represents a significant confound that undermines interpretation of both proliferation and transcriptomic effects.

**Response:** We sincerely thank the reviewer for this critical and important point. We acknowledge that the lack of an osmotic control, such as equimolar mannitol, is a major limitation of our study. It prevents us from definitively attributing the observed effects solely to hyperglycemia, as opposed to hyperosmotic stress. We have now explicitly acknowledged this limitation in the revised manuscript.

In the Discussion, we have expanded the limitations section: "Second, the use of 50 mM glucose without an equimolar osmotic control (e.g., mannitol) is a significant confound. We cannot exclude the possibility that some of the observed effects on cell proliferation, EIF1 expression, and the transcriptome may be partly attributable to hyperosmotic stress rather than the specific metabolic effects of high glucose. Future studies must include appropriate osmotic controls to dissect these distinct pathways." Page 14, Line 372-378

We have also tempered the language in the Results section (3.1) from "...suggesting that the hyperglycemic environment may impair..." to "...suggesting that exposure to high glucose conditions may impair...". Page 8, Line 220-221
-The central claim that EIF1 “drives” cell cycle dysregulation is not directly supported by functional evidence. No cell cycle profiling (e.g., flow cytometry), rescue experiments, or downstream perturbations of identified targets are provided. At present, the data demonstrate association rather than causation.

**Response:** Response: We agree completely. Our data are correlational and do not prove causation. We have revised the language throughout the manuscript to reflect this, replacing causative terms with associative ones. Key changes include:

Title: Changed from "EIF1 coordinates transcriptomic and splicing networks to drive cell cycle dysregulation..." to "EIF1 coordinates transcriptomic and splicing networks associated with cell cycle dysregulation..." Page 1, Line 1-2

Abstract: Changed "...indicating that EIF1 may regulate the cell cycle..." to "...indicating that EIF1 may be associated with cell cycle..." Page 2, Line 51-53

Discussion: Added a clear statement: "Therefore, the data demonstrate association, not causation. " and "Third, while we identified associations with gene expression and splicing, the claim that EIF1 'drives' cell cycle dysregulation is not supported by direct functional evidence such as cell cycle profiling by flow cytometry, rescue experiments, or perturbation of downstream targets. Future studies should include these functional assays to establish a causal role for EIF1. " Page 14, Line 378-382

-The alternative splicing analysis is extensive but remains purely computational. None of the highlighted splicing events (e.g., MCM7, COP1, HMGA1) are validated by independent RT-PCR with isoform-specific primers, which is a standard expectation for splicing-focused studies.

**Response:** We sincerely thank the reviewer for this critical and constructive comment. We fully agree that independent experimental validation is essential for computational predictions, especially in splicing-focused studies. In response, we have performed RT‑qPCR validation experiments for both the DEGs and the ASEs highlighted in our manuscript, using independent biological replicates and isoform‑specific primers where appropriate.

Validation results:

DEGs: All ten selected DEGs — MMP24, CYP1B1, FANCA, GINS2, MCM4, MELK, BIRC5, PLK1, RAD51, and UBE2C — were successfully validated, confirming the robustness of our RNA‑seq differential expression analysis. These results are now incorporated as a new panel Fig. 2i in the revised manuscript.

ASEs:

Successfully validated: The alternative splicing events in COP1 and TFE3 were confirmed by RT‑qPCR using isoform‑specific primers. These validated events are now presented in the main figures (Fig. 3 and Fig. 4) as core findings of our study.

Not validated: The predicted alternative splicing events in MCM7, CPSF4, and HMGA1 could not be confirmed by RT‑qPCR. To maintain transparency and scientific rigor, these unvalidated events have been moved to Supplementary Fig. S3, and the main text has been revised to focus only on the experimentally confirmed splicing events.

We are grateful to the reviewer for emphasizing the need for validation, which has allowed us to distinguish between robust and potentially false‑positive predictions. The lack of validation for MCM7, CPSF4, and HMGA1 may reflect limitations of the computational pipeline (e.g., low‑abundance isoforms, junction read thresholds) or technical challenges in primer design. By presenting only validated events in the main figures, we ensure that our conclusions are built on reliable experimental evidence.

-The manuscript frequently extrapolates findings from ARPE-19 cells to diabetic retinopathy in patients. These extrapolations should be explicitly framed as hypotheses rather than conclusions, particularly when invoking therapeutic relevance.
**Response:** We have carefully reviewed the manuscript and toned down all language that extrapolates our in vitro findings to human disease. We now frame these implications as hypotheses or suggestions for future research.

Abstract: Changed "suggesting it may be a potential therapeutic target for DR treatment." to "suggests a potential role for EIF1 in DR pathogenesis in an in vitro model, identifying it as a candidate for further investigation. " Page 2, Line 54-55

Conclusion: Changed "These discoveries... identify it as a promising molecular target for therapeutic intervention, offering substantial theoretical support for the development of precision treatments..." to "These discoveries provide a foundational framework for understanding EIF1's potential role in DR and highlight it as a candidate warranting further investigation for therapeutic targeting. " Page 15, Line 402-404

Minor concerns
-The abstract and conclusions repeatedly describe EIF1 as a “crucial” or “pivotal” regulator in diabetic retinopathy. This language is stronger than warranted by the data and should be moderated to reflect the exploratory and transcriptomic nature of the study.

**Response:** We have moderated this language as suggested. Strong claims have been replaced with more cautious terms such as "potential," "associated with," "may be involved in," and "candidate." These changes are reflected throughout the Abstract, Discussion, and Conclusion.

-The introduction would benefit from a clearer distinction between EIF1’s established role in translation initiation and the more speculative links to alternative splicing; mechanistic plausibility should be discussed more cautiously.

**Response:** We appreciate this suggestion. We have revised the Introduction to more clearly separate EIF1’s canonical function in translation initiation from its emerging, but still speculative, roles in alternative splicing and cell cycle regulation. We now frame the latter as hypotheses supported by recent literature, and we explicitly state that the mechanistic link between EIF1 and splicing in DR remains to be fully elucidated.

Changes in Manuscript:

Section 1, Page 3, Line 73-77: Revised to:

“Beyond its canonical role in translation initiation, emerging evidence suggests that EIF1 may participate in broader regulatory processes, including alternative splicing and cell cycle control, although these functions remain poorly characterized in the context of diabetic retinopathy.”

-In the Methods section, details of biological replication for RNA-seq should be stated more explicitly, including whether replicates were independent cultures and how batch effects were assessed or mitigated.

**Response:** We thank the reviewer for this request. We have added this crucial information to the Methods section (2.6).

“Three independent biological replicates were prepared for each condition (si-NC and si-EIF1). All six samples were processed in parallel for library preparation and sequenced in a single run to minimize batch effects.”Page 6, Line 153-155

-The DEG analysis uses FPKM values for visualization while DESeq2 operates on raw counts; although common, this should be clearly justified, and the normalization strategy should be described more transparently.

**Response:** We thank the reviewer for this opportunity to clarify. We have updated the Methods section (2.7) to be more transparent.

“...For visualization purposes, uniquely mapped reads were used for gene reads number counting and FPKM calculation (fragments per kilobase of transcript per million fragments mapped). All differential expression analysis was performed using DESeq2, which internally applies its own normalization procedure based on the median of ratios method, directly on the raw count data to ensure statistical rigor.” Page 6, Line 163-169

-The description of the ABLas pipeline lacks sufficient detail for reproducibility, particularly regarding versioning, parameter settings, and junction read thresholds.

**Response:** We thank the reviewer for highlighting the need for more detailed methodological description to ensure reproducibility. In response, we have substantially expanded the description of the ABLas pipeline in the Methods section, including version information, parameter settings, junction read thresholds, and statistical criteria for identifying regulated alternative splicing events (RASEs). The revised section now reads as follows:

“ For sample pair comparison, Fisher’s exact test was selected to determine statistical significance, using the alternative reads and model reads of the samples as input data. We calculated the changed ratio of alternatively spliced reads and constitutively spliced reads between compared samples, which was defined as the RASE ratio. The RASE ratio >=0.2 and p-value <= 0.05 were set as the threshold for RASEs detection. For repetition comparison, Student’s t-test was performed to evaluate the significance of the ratio alteration of AS events. Those events which were significant at P-value cutoff of 0.05 were considered non intron retention (NIR) RASEs.” Page 7, Line 179-187

-Statistical reporting is inconsistent across figures, with occasional discrepancies between stated tests and experimental design (e.g., use of t-tests where multiple comparisons may be implied).

**Response:** We have thoroughly reviewed all figures and their associated legends. We have standardized the statistical reporting. For comparisons involving only two groups, a two-tailed Student's t-test was used and is now clearly stated in the legends. For comparisons involving more than two groups, a two-way ANOVA with Tukey's post-hoc test was used and is now clearly stated. Page 8, Line 202-207

-Figure legends are generally descriptive but often repeat results rather than explaining experimental context; they should be revised to be fully interpretable without reference to the main text.

**Response:** We have revised all figure legends to provide sufficient experimental context (e.g., treatment conditions, time points, assays used) so that each figure can be interpreted independently. Redundant descriptions of results have been removed. Page 17, Line 500-582

-Several figures, particularly the splicing sashimi plots, are visually dense; increasing resolution and simplifying labeling would improve clarity.

**Response:** We have regenerated all sashimi plots at 300 dpi and simplified the labeling to improve clarity. These updated figures are now included in the revised manuscript.

-The discussion selectively cites supportive literature but does not adequately address studies that emphasize vascular endothelial cells or inflammatory pathways as primary drivers of diabetic retinopathy, which would help contextualize the RPE-centric focus.

**Response:** We thank the reviewer for this insightful suggestion. We agree that contextualizing our RPE-centric findings within the broader, multifactorial landscape of DR pathophysiology is crucial for a balanced discussion. As recommended, we have revised the opening paragraph of the Discussion to explicitly acknowledge the well-established roles of vascular endothelial cells, inflammation, and other pathways in DR, before introducing our focus on EIF1 and the RPE. This revision, as shown below, helps to frame our work as a piece of a larger puzzle rather than an isolated mechanism.

The revised text in the Discussion now reads:

"Diabetic retinopathy (DR) is one of the most severe microvascular complications of diabetes mellitus and remains incompletely understood in terms of its precise pathological mechanisms. It is now well-established that DR is a multifactorial disease involving the complex interplay of various retinal cell types, including vascular endothelial cells, pericytes, neurons, and glial cells, with pathways of inflammation, oxidative stress, and angiogenesis playing central roles. While vascular dysfunction has historically been considered the primary driver of DR, accumulating evidence highlights the critical contribution of the retinal pigment epithelium (RPE) to retinal health and its susceptibility to diabetic insult. Eukaryotic translation initiation factor EIF1, a core regulator of protein synthesis... Notably, Zhao et al. first identified a significant inverse correlation between EIF1 expression levels and glucotoxic conditions through transcriptomic analysis of high glucose-stimulated human retinal endothelial cells (hRECs), suggesting EIF1 as a potential therapeutic target for DR intervention. However, its specific regulatory network requires further elucidation."Page 11, Line 301-307

We believe this addition provides the necessary context for our study and strengthens the manuscript by presenting a more comprehensive view of the disease. Thank you for helping us improve the manuscript's balance and clarity.

-The limitations section is present but understated; additional acknowledgment of osmotic stress, lack of functional validation, and absence of in vivo confirmation would strengthen the manuscript’s credibility.

**Response:** We have significantly expanded the limitations section in the Discussion to incorporate all the points raised by the reviewer and ourselves. It now reads as follows:

“Despite the breakthrough implications of our findings for DR therapeutics, several limitations warrant consideration. First, our study was conducted exclusively in ARPE-19 cells, a transformed cell line that may not fully replicate the behavior of primary RPE cells or the complex in vivo environment of the diabetic retina. Therefore, our findings should be considered as hypothesis-generating and require validation in primary RPE cultures and in vivo animal models of DR. Second, the use of 50 mM glucose without an equimolar osmotic control (e.g., mannitol) is a significant confound. We cannot exclude the possibility that some of the observed effects on cell proliferation, EIF1 expression, and the transcriptome may be partly attributable to hyperosmotic stress rather than the specific metabolic effects of high glucose. Future studies must include appropriate osmotic controls to dissect these distinct pathways. Third, we have not provided direct functional evidence to support a causal role for EIF1. The data demonstrate association, not causation. Future studies must include cell cycle profiling (e.g., flow cytometry), rescue experiments (overexpressing EIF1 in high-glucose conditions), and perturbation of downstream targets to establish a causal and mechanistic link. Finally, the translational relevance of EIF1 as a therapeutic target remains speculative without confirmation in human samples or animal models. Nevertheless, this study establishes novel associations between EIF1 and transcriptional/splicing networks in an in vitro model of DR, providing a foundation for subsequent mechanistic investigations.” Page 14, Line 367-386

-The scientific English is generally understandable but uneven. There are recurrent issues with article usage, tense consistency, and overextended sentences that would benefit from professional language editing.
**Response:** We thank the reviewer for this feedback. We have carefully re-read the entire manuscript and have made extensive revisions to improve the English language quality. We have corrected article usage, ensured tense consistency, and broken down long, complex sentences into shorter, clearer ones. We believe these revisions have significantly improved the readability and clarity of the manuscript.

I would suggest to authors that your dataset is potentially valuable as a resource describing EIF1-associated transcriptional and splicing changes under hyperglycemic stress. With substantial revision to temper claims, improve methodological rigor, and add targeted validation, the manuscript could meet the publication standards. In its current form, however, the study does not yet provide sufficient experimental depth to support its mechanistic and translational conclusions.

**Response:** We sincerely thank the reviewer for the encouraging assessment and for recognizing the potential value of our dataset. In response to the key areas highlighted, we have made substantial revisions:

Tempering Claims: We have systematically revised the language throughout the manuscript, replacing causative terms with associative ones (e.g., "associated with," "may be involved in"). The title and conclusions now appropriately reflect the exploratory nature of the study.

Improving Methodological Rigor: We have enhanced the Methods section with detailed descriptions of biological replicates, normalization strategies, and bioinformatics parameters. We have also explicitly acknowledged the lack of an osmotic control as a limitation.

Adding Targeted Validation: As detailed above, we have performed RT-qPCR validation for both DEGs and ASEs. Ten DEGs were successfully validated, and two ASEs (COP1 and TFE3) were confirmed.

We believe that these revisions, particularly the addition of experimental validation, have substantially increased the experimental depth and credibility of the manuscript. While we acknowledge that further functional studies are needed to establish causality, we hope that the revised manuscript now meets the publication standards of PLOS ONE as a valuable resource and hypothesis-generating study.
