# Supplementary material for: EIF1 coordinates transcriptomic and splicing networks associated with cell cycle dysregulation in diabetic retinopathy
Source: PLoS One. 2026 Sep 3;21(9):e0355473. doi: 10.1371/journal.pone.0355473 (PMC13541169; doi:10.1371/journal.pone.0355473)
Supplement: Supplementary file 4 [file pone.0355473.s007.docx]

Dear Dr. Li,

Thank you for submitting your manuscript to PLOS ONE. After careful consideration, we feel that it has merit but does not fully meet PLOS ONE’s publication criteria as it currently stands. Therefore, we invite you to submit a revised version of the manuscript that addresses the points raised during the review process.

Your revised manuscript was reviewed by the four referees who had reviewed the original one. Although they found it significantly improved, they still raised some concerns. Please revise it according to their suggestions.

Please submit your revised manuscript by Jul 10 2026 11:59PM. If you will need more time than this to complete your revisions, please reply to this message or contact the journal office at plosone@plos.org. When you're ready to submit your revision, log on to https://www.editorialmanager.com/pone/ and select the 'Submissions Needing Revision' folder to locate your manuscript file.

Please include the following items when submitting your revised manuscript:

A letter that responds to each point raised by the academic editor and reviewer(s). You should upload this letter as a separate file labeled 'Response to Reviewers'.

A marked-up copy of your manuscript that highlights changes made to the original version. You should upload this as a separate file labeled 'Revised Manuscript with Track Changes'.

An unmarked version of your revised paper without tracked changes. You should upload this as a separate file labeled 'Manuscript'.

If you would like to make changes to your financial disclosure, please include your updated statement in your cover letter. Guidelines for resubmitting your figure files are available below the reviewer comments at the end of this letter.

If applicable, we recommend that you deposit your laboratory protocols in protocols.io to enhance the reproducibility of your results. Protocols.io assigns your protocol its own identifier (DOI) so that it can be cited independently in the future. For instructions see: https://journals.plos.org/plosone/s/submission-guidelines#loc-laboratory-protocols. Additionally, PLOS ONE offers an option for publishing peer-reviewed Lab Protocol articles, which describe protocols hosted on protocols.io. Read more information on sharing protocols at https://plos.org/protocols?utm_medium=editorial-email&utm_source=authorletters&utm_campaign=protocols.

As the corresponding author, your ORCID iD is verified in the submission system and will appear in the published article. PLOS supports the use of ORCID, and we encourage all coauthors to register for an ORCID iD and use it as well. Please encourage your coauthors to verify their ORCID iD within the submission system before final acceptance, as unverified ORCID iDs will not appear in the published article. Only the individual author can complete the verification step; PLOS staff cannot verify ORCID iDs on behalf of authors.

We look forward to receiving your revised manuscript.

Kind regards,

Hodaka Fujii, M.D., Ph.D.

Academic Editor

PLOS One

Journal Requirements:

If the reviewer comments include a recommendation to cite specific previously published works, please review and evaluate these publications to determine whether they are relevant and should be cited. There is no requirement to cite these works unless the editor has indicated otherwise.

[Note: HTML markup is below. Please do not edit.]

Dear Dr. Fujii and Reviewers,

We thank the academic editor and all reviewers for their constructive comments on our manuscript (PONE-D-25-49013R1). We have carefully addressed each point raised. Below we provide point-by-point responses. The corresponding revisions have been made in the marked-up copy of the manuscript (submitted as “Revised Manuscript with Track Changes”) and are also reflected in the clean version (“Manuscript”).

Reviewers' comments:

Reviewer's Responses to Questions

Comments to the Author

1. If the authors have adequately addressed your comments raised in a previous round of review and you feel that this manuscript is now acceptable for publication, you may indicate that here to bypass the “Comments to the Author” section, enter your conflict of interest statement in the “Confidential to Editor” section, and submit your "Accept" recommendation.

Reviewer #1: All comments have been addressed

Reviewer #2: (No Response)

Reviewer #3: (No Response)

Reviewer #4: All comments have been addressed

2. Is the manuscript technically sound, and do the data support the conclusions?

The manuscript must describe a technically sound piece of scientific research with data that supports the conclusions. Experiments must have been conducted rigorously, with appropriate controls, replication, and sample sizes. The conclusions must be drawn appropriately based on the data presented.

Reviewer #1: Yes

Reviewer #2: Partly

Reviewer #3: Yes

Reviewer #4: Yes

3. Has the statistical analysis been performed appropriately and rigorously?

Reviewer #1: Yes

Reviewer #2: Yes

Reviewer #3: Yes

Reviewer #4: Yes

4. Have the authors made all data underlying the findings in their manuscript fully available?

The PLOS Data policy requires authors to make all data underlying the findings described in their manuscript fully available without restriction, with rare exception (please refer to the Data Availability Statement in the manuscript PDF file). The data should be provided as part of the manuscript or its supporting information, or deposited to a public repository. For example, in addition to summary statistics, the data points behind means, medians and variance measures should be available. If there are restrictions on publicly sharing data—e.g. participant privacy or use of data from a third party—those must be specified.

Reviewer #1: Yes

Reviewer #2: Yes

Reviewer #3: Yes

Reviewer #4: Yes

5. Is the manuscript presented in an intelligible fashion and written in standard English?

PLOS ONE does not copyedit accepted manuscripts, so the language in submitted articles must be clear, correct, and unambiguous. Any typographical or grammatical errors should be corrected at revision, so please note any specific errors here.

Reviewer #1: Yes

Reviewer #2: Yes

Reviewer #3: Yes

Reviewer #4: No

6. Review Comments to the Author

Please use the space provided to explain your answers to the questions above. You may also include additional comments for the author, including concerns about dual publication, research ethics, or publication ethics. (Please upload your review as an attachment if it exceeds 20,000 characters)

Reviewer #1: This manuscript is significantly improved over the original submission. Changes have been made that clarify information in the Materials and Methods and in the Results and the Discussion that address the limitations of the study.

The English is good, although not perfect. Please note that the first sentence of the Discussion (line 299) is not a good sentence. I suggest removing the word "is" after "DR".

Note: Most writing by native English speakers is not perfect. Also, the use of articles in language can be tricky. Chinese does not use them, English does but uses them much less than French while Russian also uses articles, but uses them much less than English does.

Overall, I think that this work provides some significant information that will be useful in furthering the understanding and potentially treatment of DR.

**Response:** We are grateful for this careful reading. We have corrected the first sentence of the Discussion as recommended. The original sentence “DR is one of the most severe microvascular complications of diabetes mellitus, remains incompletely understood…” has been revised to “DR, one of the most severe microvascular complications of diabetes mellitus, remains incompletely understood…” (Line 312). Thank you again for improving our manuscript.

Reviewer #2: The revision corrects most Round 1 deficiencies. RT-qPCR confirmation of differential splicing in COP1 and TFE3 provides the experimental validation the splicing analysis required, and moving MCM7, CPSF4, and HMGA1 to supplementary figures is the correct approach given that they could not be confirmed. Statistical reporting now distinguishes SD for cellular assays from SEM for RNA-seq-derived FPKM values, with Tukey's post-hoc test specified for two-way ANOVA comparisons. Biological replication (three independent cultures per condition, sequenced in a single run) is now stated. The ARPE-19 limitation, osmotic confound from 50 mM glucose without mannitol control, and absence of functional validation are now explicitly acknowledged in the limitations section. The abstract and most of the body use appropriately hedged language framing the findings as associative and hypothesis-generating. Two concerns remain.

1. (Major) Raw RNA-seq reads have not been deposited in a public repository. The Data Availability Statement currently reads "All relevant data are within the manuscript and its Supporting Information files." Primary sequencing reads cannot be embedded in a manuscript; PLOS ONE data policy requires deposition in NCBI GEO or SRA prior to acceptance, with the accession number provided in the Data Availability Statement and cited in the Methods. No GEO or SRA accession number appears anywhere in the submission. The authors must deposit the raw reads, generate an accession number, and update the Data Availability Statement and Methods accordingly before this manuscript can be accepted.

**Response:** We thank the reviewer for pointing out this critical omission. We have now deposited the raw RNA-seq reads in the NCBI Gene Expression Omnibus (GEO) under accession number GSE334496. We have updated the Data Availability Statement in the manuscript. The revised Data Availability Statement reads (Line 415-417): “The raw RNA-seq data generated in this study have been deposited in the NCBI Gene Expression Omnibus (GEO) under accession number GSE334496.” We are grateful for this essential guidance.

2. (Minor) The Discussion closing passages retain overclaiming language that was not corrected. Line 331 places EIF1 as "a pivotal molecular mediator linking hyperglycemia to cell cycle dysregulation in DR." Line 367 opens the limitations paragraph with "Despite the breakthrough implications of our findings for DR therapeutics." Lines 387-391 state the work "elucidates pivotal connections" and holds "substantial translational value...offering innovative perspectives that could reshape future treatment paradigms." These passages are inconsistent with the moderated abstract and with a study constrained to a single transformed cell line, lacking an osmotic control, and providing no causal functional evidence. The language should be brought to the same epistemic level as the rest of the revised manuscript. The same section also contains a grammatical error at line 295-296 ("mechanistically may implicating their dysregulation") that persists despite the stated language revision.

**Response:** We thank the reviewer for this important reminder to maintain appropriate scientific caution. We have toned down the overclaiming language throughout the Discussion and Conclusion sections. Specific changes are as follows:

- Line 343-345: “These results position EIF1 as a pivotal molecular mediator linking hyperglycemia to cell cycle dysregulation in DR” → Revised: “These results suggest that EIF1 may be associated with cell cycle dysregulation in DR under hyperglycemic conditions”.
- Line 383-384: “Despite the breakthrough implications of our findings for DR therapeutics” → Revised: “Although our findings suggest potential relevance for understanding DR pathogenesis”.
- Grammatical error (Line 308): “mechanistically may implicating their dysregulation” → Revised: “potentially implicating their dysregulation”.

We are grateful for the reviewer’s meticulous attention to language.

Reviewer #3: This study investigates the role of eukaryotic translation initiation factor 1 (EIF1) in diabetic retinopathy (DR). Using an in vitro model of high glucose-treated human retinal pigment epithelial cells (ARPE-19), we observed that high glucose levels significantly inhibited cell proliferation, a phenomenon associated with EIF1 downregulation. This suggests that EIF1 depletion induced by high glucose may impair cellular function. Transcriptome sequencing revealed that EIF1 knockdown co-induced 223 differentially expressed genes, with 188 of these downregulated genes being significantly enriched in cell cycle-related pathways. Concurrently, 1211 differential alternative splicing events were detected, primarily impacting the cell cycle and p53 signaling pathway. The key differentially expressed genes and splicing pattern changes of COP1 and TFE3 were validated using RT-qPCR. These findings suggest that EIF1 may contribute to cell cycle dysregulation in DR by regulating gene expression and alternative splicing, thereby laying the groundwork for future mechanistic investigations.

1.In the abstract and introduction sections, it is recommended to further clarify the distinction between EIF1's roles in translation initiation and RNA binding functions to avoid conflating these two processes.

**Response:** We thank the reviewer for this important suggestion to avoid conflating EIF1’s distinct functional roles. In response, we have revised the Introduction section to more clearly distinguish between EIF1’s canonical function in translation initiation and its capacity as an RNA-binding protein. The revised text now reads (Line 71-78): “Eukaryotic translation initiation factor 1 (EIF1; aliases: A121, ISO1, SUI1, EIF-1, EIF1A), a 12-kDa protein that functions as a critical mediator of start codon recognition during eukaryotic translation initiation[7-8]. Beyond its canonical role in translation initiation[9], EIF1 has been classified as an RNA-binding protein (RBP) based on its ability to interact with RNA molecules, and emerging evidence suggests that it may participate in...” This revision first establishes EIF1’s primary functional identity as a translation initiation factor, then separately introduces its RNA-binding capacity as a basis for potential broader regulatory roles, thus drawing a clear conceptual distinction between these two aspects. We are grateful for the reviewer’s careful reading and constructive suggestion.

2. The following references are closely related to the authors' research topic and should be cited:

[1] Yan Z-H, Zhao D-M, Wang X-T, et al. “Medicine food homology” plants in the treatment of diabetic nephropathy: pathogenic pathways and therapeutic approaches. Food & Medicine Homology, 2025, https://doi.org/10.26599/FMH.2026.9420115

[2] Asare, A., Adjei Broni, A., Asare Dickson, A.K., Sagoe, M. and Cudjoe, J.M. (2025), Performance of ResNet-18 and InceptionResNetV2 in Automated Detection of Diabetic Retinopathy. Med. Adv, 3: 231-241. https://doi.org/10.1002/med4.70023

[3] Li M-Y, Wu G-F, Lu F, et al. Food and Medicine Homology Focus in 2026. Food & Medicine Homology, 2026, 3(2): 9420133. <https://doi.org/10.26599/FMH.2026.9420133>

**Response:** We thank the reviewer for providing these references, which we have carefully reviewed.

Regarding references [1] and [3]: After thorough evaluation, we note that reference [1] focuses on diabetic nephropathy (not diabetic retinopathy) and the therapeutic application of “medicine-food homology” plants, while reference [3] is a perspective article on the field of food and medicine homology. Neither directly addresses the molecular mechanisms of diabetic retinopathy or the role of RNA-binding proteins such as EIF1 in this disease. Therefore, we respectfully conclude that references [1] and [3] are not directly relevant to our study and will not be cited.

Regarding reference [2] (Asare A et al., Performance of ResNet-18 and InceptionResNetV2 in Automated Detection of Diabetic Retinopathy, Med. Adv, 2025, 3: 231-241): This paper provides valuable background on the epidemiology of diabetic retinopathy (DR), noting that DR affects approximately 34.6% of diabetic individuals and is a leading cause of vision loss. Our manuscript presents similar epidemiological data (30–40% prevalence, over 100 million affected globally). Therefore, we have added reference [2] as an additional citation to support the existing epidemiological statements in the Introduction.

We thank the reviewer for bringing this relevant reference to our attention.

3.The description of cell cycle-related genes in the discussion section appears overly list-like; it is suggested to strengthen the functional connections among these genes and their synergistic effects in differentiation-related (DR) processes.

**Response:** We thank the reviewer for this constructive suggestion to strengthen the functional connections among the cell cycle-related genes and their synergistic effects in DR. In the Discussion section, we have substantially revised the paragraph describing these DEGs. Instead of presenting them as an isolated list, we have now reorganized the text by functional modules—DNA replication initiation (MCM4 and GINS2), mitotic progression (PLK1 and MELK), DNA repair (RAD51 and FANCA), and apoptosis/mitotic coordination (BIRC5)—while retaining the specific literature evidence for each gene in diabetic or hyperglycemic contexts (Line 350-363). We are grateful for the reviewer's insightful suggestion to improve the scientific narrative.

4.For unverified alternative splicing (AS) events, potential technical reasons should be briefly explained in the main text.

**Response:** We thank the reviewer for this suggestion to provide technical explanations for the unverified alternative splicing events. In section 3.3, we have added a brief explanation addressing this issue. The revised text now reads (Line 300-306): “Unfortunately, the remaining three candidate AS events (MCM7, CPSF4, and HMGA1) did not show consistent splicing changes by RT-qPCR. This discrepancy may be attributed to the inherent limitations of computational prediction from short-read RNA-seq data, which can yield false-positive AS events, or to relatively low abundance of the spliced isoforms that falls below the reliable detection threshold of qPCR.” This explanation covers two potential technical reasons: (1) the inherent limitations of short-read RNA-seq-based computational prediction, which can generate false-positive calls, and (2) the possibility that the spliced isoforms are present at low abundance, below the reliable detection limit of qPCR. We are grateful for the reviewer's suggestion to improve the transparency of our result presentation.

5. In the conclusion section, strong statements such as " should be revised to more moderate expressions like "laying a foundation for subsequent studies" or "suggesting their potential value."

**Response:** We thank the reviewer for this important reminder to maintain appropriate scientific restraint. The Conclusion section has been rewritten to be more moderate. The revised conclusion states (Line 407-410): “In summary, this study suggests that EIF1 is downregulated under high-glucose conditions and may regulate the expression and alternative splicing of cell cycle-related genes in ARPE-19 cells. These findings point to EIF1 as a candidate worthy of further investigation in DR pathogenesis.” We appreciate the reviewer’s emphasis on accurate scientific communication.

6. In the discussion section, the mechanism by which EIF1 simultaneously regulates transcription and splicing needs clarification regarding whether this involves direct or indirect actions, to prevent mechanistic confusion when presenting these two functions side by side.

**Response:** We thank the reviewer for this important mechanistic question. We acknowledge that, as an RNA-binding protein, EIF1 possesses the potential to directly interact with RNA targets. However, based on our current transcriptomic data alone, we are unable to determine whether the observed changes in gene expression and splicing arise from EIF1's direct binding to specific RNA targets, or indirectly through its canonical function in translation initiation—for instance, via translational regulation of key transcription or splicing factors that in turn affect downstream networks. Additionally, comprehensive identification of EIF1's genome-wide RNA binding targets in the context of DR has not yet been performed. These uncertainties together preclude a definitive mechanistic assignment at this stage. In response, we have added a brief statement in the limitations paragraph of the Discussion to address this issue. The revised text now reads (Line 394-400): “Third, our data demonstrate association rather than causation, and we cannot yet distinguish whether EIF1 regulates downstream gene expression or splicing through direct RNA binding or indirectly via downstream effectors.. Future studies CLIP-seq or iRIP-seq to map EIF1’s direct RNA targets, along with cell cycle profiling, rescue experiments, and perturbation of downstream candidates, will be required to establish both the direct-versus-indirect mechanisms and the causal role of EIF1 in DR pathogenesis. ”We are grateful for the reviewer's thoughtful suggestion to prevent mechanistic confusion.

7. For genes with validated splicing events such as COP1 and TFE3, the possible functional differences of their splice variants should be briefly discussed in the main text to enhance the logical connection with cell cycle regulation.

**Response:** We thank the reviewer for this constructive suggestion to strengthen the logical connection between EIF1-mediated alternative splicing and cell cycle regulation. In the Discussion section, we have used cautious, hypothetical language throughout, as we lack direct experimental evidence that these splicing events generate functionally distinct proteins. We have revised the concluding sentence of this paragraph to more clearly articulate our hypothesis (Line 377-380): “Based on these observations, we hypothesize that EIF1-mediated alternative splicing of COP1 and TFE3 may generate functionally distinct protein isoforms that contribute to cell cycle dysregulation in DR.” This revision explicitly frames our proposal as a hypothesis rather than a conclusion, while establishing a clear logical chain linking EIF1-mediated splicing, generation of distinct protein isoforms, and dysregulation of cell cycle in DR. We are grateful for the reviewer's insightful suggestion to clarify these mechanistic connections.

8. It is recommended to standardize the terminology for "cell cycle dysregulation" throughout the manuscript, avoiding inconsistent use of terms like "abnormal," "disordered," or "dysregulated" in different chapters to improve terminological consistency.

**Response:** We thank the reviewer for this attention to terminological consistency. We have carefully re-examined the entire manuscript and confirm that there are no instances of "abnormal cell cycle," "disordered cell cycle," or other inconsistent variants. All descriptions of cell cycle abnormalities uniformly use "cell cycle dysregulation" (or "dysregulation of the cell cycle"). Therefore, no further changes are needed on this point. We are grateful for the reviewer's careful reading and for helping us maintain terminological rigor.

Reviewer #4: Reviewer Comments

This study focuses on the molecular mechanism of EIF1 regulating the cell cycle in diabetic retinopathy (DR) using a classic research approach combining RNA-seq with functional validation. The research topic aligns with PLOS ONE’s focus on basic medical and molecular mechanism research. The experimental design is basically rational, the data are complete, and most of the requirements for the first round of revision have been met.

1. General Evaluation

Using ARPE-19 cells as an in vitro model, this study investigated the expression changes and functions of EIF1 in high glucose-induced DR, and found that EIF1 knockdown leads to cell cycle dysregulation by regulating the differential expression and alternative splicing of cell cycle-related genes. It is the first to reveal the potential role of EIF1 in the functional abnormalities of retinal pigment epithelial cells in DR, providing new clues for the study of the molecular mechanism of DR. The authors have completed key revisions in accordance with the first round of reviewer comments, including weakening causal conclusions, supplementing experimental validation, improving methodological details, and expanding research limitations, which have greatly enhanced the scientificity and rigor of the manuscript. At present, the manuscript still has some minor details to be further improved, with no defects in core scientific issues, and it is recommended to accept after minor revision.

2.Specific Revision Suggestions

（1）Supplementary Methodological Details

Description of cell experimental groups: In 2.3 Cell proliferation assay, only 0, 30, and 50 mM glucose groups are mentioned, and the glucose concentration of the "normal control group" (routinely 5.5 mM) is not specified. It is recommended to supplement this basic information to standardize the experimental grouping and facilitate readers to understand the basis for setting the concentration gradient of high glucose treatment.

**Response:** We thank the reviewer for this important suggestion to improve methodological clarity. In section 2.3, we have specified the glucose concentration of the normal control group. The revised text now reads (Line 111): “experimental groups treated with 30 mM or 50 mM glucose and normal controls containing culture medium only (5.5 mM glucose)” , which clarifies the basis for setting the high glucose concentration gradients (30 mM and 50 mM). We are grateful for the reviewer's careful attention to experimental detail.

（2）RT-qPCR primer information: The manuscript mentions that primer information is shown in Supplementary Table S1, but it is not clearly stated in the manuscript whether Table S1 contains core information such as primer sequences, amplified fragment lengths, and annealing temperatures. It is recommended to confirm the completeness of this information in the description of supplementary materials to meet the repeatability requirements of molecular experiments.

**Response:** We thank the reviewer for the suggestion to provide more detailed RT-qPCR methodology. In section 2.4, we have added: (1) the instrument model (ABI QuantStudio 5), (2) the thermal cycling program, (3) technical replicates (triplicate), (4) a detailed description of the primer design strategy and calculation method for validating alternative splicing events, and (5) a clear statement that primer sequences, amplicon lengths are provided in Supplementary Table S1 (Line 120-129).

（3）Quantification description of protein detection: Section 2.5 Western blot only describes the experimental procedure, without specifying the quantification method (e.g., gray value analysis) and software used for protein bands. It is recommended to supplement this detail to make the analysis of protein experimental results more operable.

**Response:** We thank the reviewer for the suggestion to improve the methodological description of protein quantification. In section 2.5, we have added the following details: “Protein bands were quantified by densitometry using ImageJ software (NIH, USA), and band intensities were normalized to GAPDH.” Line 144-145

3.Optimization of Result Presentation and Figures/Tables

（1）Consistency of result data presentation: There are inconsistent formats of statistical significance labels in some results (e.g., ****p<0.0001 in Fig 1, and p<0.0001 in some main text). It is recommended to unify the format of statistical significance labels throughout the manuscript (including figure legends and main text) to maintain standardization.

**Response:** We thank the reviewer for this careful observation. We have unified the format. Throughout the manuscript, figure legends and main text now consistently use: *p < 0.05; **p < 0.01; ***p < 0.001; ****p < 0.0001. We are grateful for this attention to detail.

（2）Simplification of figure legends and labels: The annotation of enrichment analysis bubble plots in some supplementary figures (e.g., Fig S2) is slightly dense, and the font of some pathway names is too small. It is recommended to appropriately simplify the legend annotation and enlarge the core pathway names to improve the readability of the figures; at the same time, confirm that the sample size (n value) of all figures is clearly marked in the figure legends without omission.

**Response:** We thank the reviewer for these constructive suggestions to improve figure readability. We have taken the following actions:

(1) Supplementary Fig. S2 readability: The enrichment analysis bubble plot in Supplementary Fig. S2 has been regenerated with enlarged font sizes for the pathway names.

(2) Sample size (n value) marking: We have carefully reviewed all figure legends and confirmed that the sample size (n=3 per group) has been uniformly and clearly marked in every figure legend where quantitative data are presented. This includes all main figures (Fig. 1-5) and supplementary figures (Fig. S1-S3).

We are grateful for the reviewer's careful attention to figure presentation and reproducibility.

（3）Presentation of alternative splicing results: Section 3.3 mentions that 5 alternative splicing-related genes were screened out, and only COP1 and TFE3 were validated, with the remaining 3 moved to supplementary materials. It is recommended to mark in the main text that "the splicing changes of other candidate genes were not verified by experiments, see Supplementary Materials" to make the result presentation more rigorous and avoid misunderstanding by readers.

**Response:** We thank the reviewer for this suggestion to improve the rigor of result presentation. In section 3.3, we have revised the text to clearly state that the remaining three candidate AS events (MCM7, CPSF4, and HMGA1) did not show consistent splicing changes by RT-qPCR. The revised sentence now reads (Line 300-306): “Unfortunately, the remaining three candidate AS events (MCM7, CPSF4, and HMGA1) did not show consistent splicing changes by RT-qPCR. This discrepancy may be attributed to the inherent limitations of computational prediction from short-read RNA-seq data, which can yield false-positive AS events, or to relatively low abundance of the spliced isoforms that falls below the reliable detection threshold of qPCR. These unconfirmed events are presented in Supplementary Fig. S3 for completeness.” We believe this revision accurately reflects the experimental outcome, appropriately acknowledges the technical limitations of both RNA-seq prediction and qPCR validation, and ensures that readers are not misled into believing that all five candidates were experimentally confirmed. The change is highlighted in bold in the revised manuscript. We are grateful for the reviewer's careful attention to scientific rigor.

4.Improvement of Discussion and Conclusions

（1）Appropriate elaboration of research significance: The discussion section has fully explained the research limitations, and the preliminary value of this study can be appropriately supplemented, such as "This study is the first to associate EIF1 with the cell cycle regulation of retinal pigment epithelial cells in DR, providing candidate molecules and transcriptomic data support for the subsequent exploration of new targets for DR", to achieve a better balance in the discussion and avoid the research significance being less prominent due to overemphasis on limitations.

**Response:** We thank the reviewer for this constructive suggestion to better balance the discussion between research significance and limitations. In response, we have added a new paragraph in the Discussion section, positioned immediately after the limitations paragraph, to appropriately highlight the preliminary value of our study. The added sentence reads: “This study is the first to associate EIF1 with cell cycle regulation in retinal pigment epithelial cells under hyperglycemic conditions, providing candidate molecules and transcriptomic data that may guide subsequent exploration of novel therapeutic targets for DR.” Line 402-405

To avoid redundancy and maintain conciseness, we have removed the subsequent “In conclusion…” transitional paragraph at the end of the Discussion, as its content is now sufficiently covered by the newly added significance statement and the independent Conclusion section (Section 5). This adjustment ensures a clear division of labor: the Discussion ends with the research significance, while Section 5 provides a comprehensive summary of the study’s findings. We are grateful for the reviewer's thoughtful suggestion to achieve better balance in the discussion.

（2）Optimization of the conciseness of conclusions: Section 5 Conclusions is slightly lengthy and can be appropriately streamlined to highlight the core findings (EIF1 is downregulated under high glucose, regulates the expression and splicing of cell cycle-related genes, and provides new candidates for DR research), delete repetitive expressions, and make the conclusions more concise in line with PLOS ONE’s writing requirements for clarity and conciseness.

**Response:** We thank the reviewer for this suggestion to streamline the Conclusions section. In response, we have significantly condensed Section 5 (Conclusions) to highlight only the core findings while removing redundant expressions. In addition, we have taken care to use appropriately cautious language to avoid overstatement. The revised conclusion now reads (Line407-410): “In summary, this study suggests that EIF1 is downregulated under high-glucose conditions and may regulate the expression and alternative splicing of cell cycle-related genes in ARPE-19 cells. These findings point to EIF1 as a candidate worthy of further investigation in DR pathogenesis.” Compared to the original version, this revision is more concise and uses more measured language (e.g., “suggests” rather than “demonstrates”), aligning with the requirement for scientific humility and PLOS ONE’s standards for clarity and conciseness.

5.Correction of Format and Text Details

（1）English spelling and punctuation: Check the English spelling and punctuation throughout the manuscript, e.g., "Refferences" should be corrected to "References", and the punctuation separation (e.g., commas and semicolons) in some long sentences needs to be further standardized to avoid affecting the manuscript quality due to minor format errors.

（2）First annotation of abbreviations: Some abbreviations in the manuscript (e.g., ASEs, RASEs, RASGs) have been annotated at their first occurrence, but it is recommended to supplement the full spelling at their first occurrence in the Abstract (e.g., alternative splicing events, ASEs) to facilitate independent reading of the abstract.

（3）Reference format: Check the consistency of reference formats, e.g., different annotation methods for volume, issue and page numbers of some literatures. It is recommended to unify the format in accordance with PLOS ONE’s reference requirements to ensure compliance with the journal typesetting specifications.

**Response:** We thank the reviewer for carefully identifying these formatting issues.

(1) English spelling and punctuation: We have corrected the spelling error “Refferences” to “References” throughout the manuscript. We have also reviewed the entire text and standardized punctuation in long sentences to improve readability.

(2) First annotation of abbreviations: We have confirmed that ASEs (alternative splicing events) is already annotated at its first occurrence in the Abstract. RASEs and RASGs do not appear in the Abstract; they first appear in the main text and have been properly annotated there. No additional changes are needed.

(3) Reference format: We have carefully revised all references according to PLOS ONE’s requirements.

We are grateful for the reviewer's meticulous attention to these formatting details.

7. PLOS authors have the option to publish the peer review history of their article (what does this mean?). If published, this will include your full peer review and any attached files.

If you choose “no”, your identity will remain anonymous but your review may still be made public.

Do you want your identity to be public for this peer review? For information about this choice, including consent withdrawal, please see our Privacy Policy.

Reviewer #1: No

Reviewer #2: No

Reviewer #3: No

Reviewer #4: No

[NOTE: If reviewer comments were submitted as an attachment file, they will be attached to this email and accessible via the submission site. Please log into your account, locate the manuscript record, and check for the action link "View Attachments". If this link does not appear, there are no attachment files.]

To ensure your figures meet our technical requirements, please review our figure guidelines: https://journals.plos.org/plosone/s/figures

You may also use PLOS’s free figure tool, NAAS, to help you prepare publication quality figures: https://journals.plos.org/plosone/s/figures#loc-tools-for-figure-preparation.

NAAS will assess whether your figures meet our technical requirements by comparing each figure against our figure specifications.

为了确保您的图表符合我们的技术要求，请查阅我们的图表指南：https://journals.plos.org/plosone/s/figures

您也可以使用PLOS的免费图表工具NAAS来帮助您准备符合出版质量的图表：https://journals.plos.org/plosone/s/figures#loc-tools-for-figure-preparation。

NAAS 将通过将您的每个图表与我们的图表规格进行比较来评估其是否符合我们的技术要求。

In compliance with data protection regulations, you may request that we remove your personal registration details at any time. (Remove my information/details). Please contact the publication office if you have any questions.
