# Supplementary material for: EIF1 coordinates transcriptomic and splicing networks associated with cell cycle dysregulation in diabetic retinopathy
Source: PLoS One. 2026 Sep 3;21(9):e0355473. doi: 10.1371/journal.pone.0355473 (PMC13541169; doi:10.1371/journal.pone.0355473)
Supplement: Peer review history

# Peer review: Decision Letter 0

**DOI:** 10.1371/journal.pone.0355473.r001

**Authors:** Kangkang Ji

30 Jan 2026

Dear Dr. Li,

Please submit your revised manuscript by Mar 16 2026 11:59PM. If you will need more time than this to complete your revisions, please reply to this message or contact the journal office at plosone@plos.org. When you're ready to submit your revision, log on to https://www.editorialmanager.com/pone/ and select the 'Submissions Needing Revision' folder to locate your manuscript file.

- A letter that responds to each point raised by the academic editor and reviewer(s). You should upload this letter as a separate file labeled 'Response to Reviewers'.
- A marked-up copy of your manuscript that highlights changes made to the original version. You should upload this as a separate file labeled 'Revised Manuscript with Track Changes'.
- An unmarked version of your revised paper without tracked changes. You should upload this as a separate file labeled 'Manuscript'.

If you would like to make changes to your financial disclosure, please include your updated statement in your cover letter. Guidelines for resubmitting your figure files are available below the reviewer comments at the end of this letter.

If applicable, we recommend that you deposit your laboratory protocols in protocols.io to enhance the reproducibility of your results. Protocols.io assigns your protocol its own identifier (DOI) so that it can be cited independently in the future. For instructions see: https://journals.plos.org/plosone/s/submission-guidelines#loc-laboratory-protocols. Additionally, PLOS ONE offers an option for publishing peer-reviewed Lab Protocol articles, which describe protocols hosted on protocols.io. Read more information on sharing protocols at https://plos.org/protocols?utm_medium=editorial-email&utm_source=authorletters&utm_campaign=protocols.

We look forward to receiving your revised manuscript.

Kind regards,

Kangkang Ji, Ph.D., M.D.

Academic Editor

PLOS One

Journal Requirements:

When submitting your revision, we need you to address these additional requirements.

1. Please ensure that your manuscript meets PLOS ONE's style requirements, including those for file naming. The PLOS ONE style templates can be found at

https://journals.plos.org/plosone/s/file?id=wjVg/PLOSOne_formatting_sample_main_body.pdf and

https://journals.plos.org/plosone/s/file?id=ba62/PLOSOne_formatting_sample_title_authors_affiliations.pdf

2. Please note that PLOS One has specific guidelines on code sharing for submissions in which author-generated code underpins the findings in the manuscript. In these cases, we expect all author-generated code to be made available without restrictions upon publication of the work. Please review our guidelines at https://journals.plos.org/plosone/s/materials-and-software-sharing#loc-sharing-code and ensure that your code is shared in a way that follows best practice and facilitates reproducibility and reuse.

3. Please update your submission to use the PLOS LaTeX template. The template and more information on our requirements for LaTeX submissions can be found at http://journals.plos.org/plosone/s/latex.

4. Thank you for stating the following in the Acknowledgments Section of your manuscript:

“This study was supported by the Natural Science Foundation of Ningxia (Grant No. [2022AAC03564])”

We note that you have provided funding information that is not currently declared in your Funding Statement. However, funding information should not appear in the Acknowledgments section or other areas of your manuscript. We will only publish funding information present in the Funding Statement section of the online submission form.

Please remove any funding-related text from the manuscript and let us know how you would like to update your Funding Statement. Currently, your Funding Statement reads as follows:

“This study was supported by the Natural Science Foundation of Ningxia (Grant No. [2022AAC03564]).”

Please include your amended statements within your cover letter; we will change the online submission form on your behalf.

5. We note that the grant information you provided in the ‘Funding Information’ and ‘Financial Disclosure’ sections do not match.

When you resubmit, please ensure that you provide the correct grant numbers for the awards you received for your study in the ‘Funding Information’ section.

6. Thank you for stating the following financial disclosure:

“This study was supported by the Natural Science Foundation of Ningxia (Grant No. [2022AAC03564]).”

Please state what role the funders took in the study.  If the funders had no role, please state: "The funders had no role in study design, data collection and analysis, decision to publish, or preparation of the manuscript."

If this statement is not correct you must amend it as needed.

Please include this amended Role of Funder statement in your cover letter; we will change the online submission form on your behalf.

7. PLOS ONE now requires that authors provide the original uncropped and unadjusted images underlying all blot or gel results reported in a submission’s figures or Supporting Information files. This policy and the journal’s other requirements for blot/gel reporting and figure preparation are described in detail at https://journals.plos.org/plosone/s/figures#loc-blot-and-gel-reporting-requirements and https://journals.plos.org/plosone/s/figures#loc-preparing-figures-from-image-files. When you submit your revised manuscript, please ensure that your figures adhere fully to these guidelines and provide the original underlying images for all blot or gel data reported in your submission. See the following link for instructions on providing the original image data: https://journals.plos.org/plosone/s/figures#loc-original-images-for-blots-and-gels.

In your cover letter, please note whether your blot/gel image data are in Supporting Information or posted at a public data repository, provide the repository URL if relevant, and provide specific details as to which raw blot/gel images, if any, are not available. Email us at plosone@plos.org if you have any questions.

8. PLOS requires an ORCID iD for the corresponding author in Editorial Manager on papers submitted after December 6th, 2016. Please ensure that you have an ORCID iD and that it is validated in Editorial Manager. To do this, go to ‘Update my Information’ (in the upper left-hand corner of the main menu), and click on the Fetch/Validate link next to the ORCID field. This will take you to the ORCID site and allow you to create a new iD or authenticate a pre-existing iD in Editorial Manager.

9. If the reviewer comments include a recommendation to cite specific previously published works, please review and evaluate these publications to determine whether they are relevant and should be cited. There is no requirement to cite these works unless the editor has indicated otherwise.

[Note: HTML markup is below. Please do not edit.]

Reviewers' comments:

Reviewer's Responses to Questions

**Comments to the Author**

1. Is the manuscript technically sound, and do the data support the conclusions?

Reviewer #1: Yes

Reviewer #2: Yes

**********

2. Has the statistical analysis been performed appropriately and rigorously?

Reviewer #1: I Don't Know

Reviewer #2: Yes

**********

3. Have the authors made all data underlying the findings in their manuscript fully available?

The PLOS Data policy

Reviewer #1: Yes

Reviewer #2: Yes

**********

4. Is the manuscript presented in an intelligible fashion and written in standard English?

Reviewer #1: Yes

Reviewer #2: Yes

**********

Reviewer #1: Overall this is a well-written paper that presents some important results. There are a few mostly minor things:

1) Western in western blotting should not be capitalized. Southern in Southern blotting is capitalized since the technique was invented by Dr. Southern. Northern blotting and western blotting were so named as a kind of scientific joke (not invented by a Dr. Northern and Dr. Western). This is not a rare error in scientific writing.

2) In the Cell Proliferation assay, I would change the last sentence to say the "The net cell proliferation rate..." rather than "The cell proliferation rate..." since the CCK-8 assay results are proportional to the number of viable cells and therefore cannot distinguish whether there was just a change in in proliferation or if cell death was also involved.

3) For evaluating RNA integrity before RNA sequencing, calculating an RNA integrity number would have been preferable to just using the A260/280 ration and (visually?) examining RNA gels.

4) In the Statistical analysis section of Materials and methods, did the authors mean to say that the data was presented as the mean plus and minus the mean sample standard deviation or the standard error of the mean? This should be reworded for clarity. I would note that it is generally more appropriate to show the mean plus or minus the standard error of the mean (SEM). I would also indicate sample sizes in the figure legends, where appropriate. Where a 2-way ANOVA was significant, what method was used for subsequent multiple comparisons?

Reviewer #2: The study reports that high-glucose treatment suppresses EIF1 expression in ARPE-19 cells and that EIF1 knockdown under hyperglycemic conditions leads to widespread changes in gene expression and alternative splicing, with enrichment in cell cycle-related pathways. The authors propose that EIF1 coordinates transcriptional and splicing networks that contribute to cell cycle dysregulation in diabetic retinopathy and suggest EIF1 as a potential therapeutic target.

I have a few major concerns and suggestions as following.

-The biological model is limited to a single transformed retinal pigment epithelial cell line (ARPE-19). While commonly used, this line is known to differ substantially from primary RPE in cell cycle behavior, metabolic responses, and differentiation status. Claims regarding diabetic retinopathy pathogenesis should therefore be substantially tempered, or additional validation in primary RPE cells or in vivo models should be included.

-The use of 50 mM glucose as the primary experimental condition raises concerns about osmotic stress rather than physiological hyperglycemia. The absence of an osmotic control (e.g., mannitol) represents a significant confound that undermines interpretation of both proliferation and transcriptomic effects.

-The central claim that EIF1 “drives” cell cycle dysregulation is not directly supported by functional evidence. No cell cycle profiling (e.g., flow cytometry), rescue experiments, or downstream perturbations of identified targets are provided. At present, the data demonstrate association rather than causation.

-The alternative splicing analysis is extensive but remains purely computational. None of the highlighted splicing events (e.g., MCM7, COP1, HMGA1) are validated by independent RT-PCR with isoform-specific primers, which is a standard expectation for splicing-focused studies.

-The manuscript frequently extrapolates findings from ARPE-19 cells to diabetic retinopathy in patients. These extrapolations should be explicitly framed as hypotheses rather than conclusions, particularly when invoking therapeutic relevance.

Minor concerns

-The abstract and conclusions repeatedly describe EIF1 as a “crucial” or “pivotal” regulator in diabetic retinopathy. This language is stronger than warranted by the data and should be moderated to reflect the exploratory and transcriptomic nature of the study.

-The introduction would benefit from a clearer distinction between EIF1’s established role in translation initiation and the more speculative links to alternative splicing; mechanistic plausibility should be discussed more cautiously.

-In the Methods section, details of biological replication for RNA-seq should be stated more explicitly, including whether replicates were independent cultures and how batch effects were assessed or mitigated.

-The DEG analysis uses FPKM values for visualization while DESeq2 operates on raw counts; although common, this should be clearly justified, and the normalization strategy should be described more transparently.

-The description of the ABLas pipeline lacks sufficient detail for reproducibility, particularly regarding versioning, parameter settings, and junction read thresholds.

-Statistical reporting is inconsistent across figures, with occasional discrepancies between stated tests and experimental design (e.g., use of t-tests where multiple comparisons may be implied).

-Figure legends are generally descriptive but often repeat results rather than explaining experimental context; they should be revised to be fully interpretable without reference to the main text.

-Several figures, particularly the splicing sashimi plots, are visually dense; increasing resolution and simplifying labeling would improve clarity.

-The discussion selectively cites supportive literature but does not adequately address studies that emphasize vascular endothelial cells or inflammatory pathways as primary drivers of diabetic retinopathy, which would help contextualize the RPE-centric focus.

-The limitations section is present but understated; additional acknowledgment of osmotic stress, lack of functional validation, and absence of in vivo confirmation would strengthen the manuscript’s credibility.

-The scientific English is generally understandable but uneven. There are recurrent issues with article usage, tense consistency, and overextended sentences that would benefit from professional language editing.

I would suggest to authors that your dataset is potentially valuable as a resource describing EIF1-associated transcriptional and splicing changes under hyperglycemic stress. With substantial revision to temper claims, improve methodological rigor, and add targeted validation, the manuscript could meet the publication standards. In its current form, however, the study does not yet provide sufficient experimental depth to support its mechanistic and translational conclusions.

**********

what does this mean?). If published, this will include your full peer review and any attached files.

If you choose “no”, your identity will remain anonymous but your review may still be made public.

**Do you want your identity to be public for this peer review?**  For information about this choice, including consent withdrawal, please see our Privacy Policy

Reviewer #1: No

Reviewer #2: No

**********

[NOTE: If reviewer comments were submitted as an attachment file, they will be attached to this email and accessible via the submission site. Please log into your account, locate the manuscript record, and check for the action link "View Attachments". If this link does not appear, there are no attachment files.]

To ensure your figures meet our technical requirements, please review our figure guidelines: https://journals.plos.org/plosone/s/figures

You may also use PLOS’s free figure tool, NAAS, to help you prepare publication quality figures: https://journals.plos.org/plosone/s/figures#loc-tools-for-figure-preparation.

NAAS will assess whether your figures meet our technical requirements by comparing each figure against our figure specifications.

# Author comment: Author response to Decision Letter 1

**DOI:** 10.1371/journal.pone.0355473.r002

15 Mar 2026

PONE-D-25-49013

EIF1 coordinates transcriptomic and splicing networks to drive cell cycle dysregulation in diabetic retinopathy

PLOS One

Dear Dr. Li,

Thank you for submitting your manuscript to PLOS ONE. After careful consideration, we feel that it has merit but does not fully meet PLOS ONE’s publication criteria as it currently stands. Therefore, we invite you to submit a revised version of the manuscript that addresses the points raised during the review process.

Please submit your revised manuscript by Mar 16 2026 11:59PM. If you will need more time than this to complete your revisions, please reply to this message or contact the journal office at plosone@plos.org. When you're ready to submit your revision, log on to https://www.editorialmanager.com/pone/ and select the 'Submissions Needing Revision' folder to locate your manuscript file.

Please include the following items when submitting your revised manuscript:

�A letter that responds to each point raised by the academic editor and reviewer(s). You should upload this letter as a separate file labeled 'Response to Reviewers'.

�A marked-up copy of your manuscript that highlights changes made to the original version. You should upload this as a separate file labeled 'Revised Manuscript with Track Changes'.

�An unmarked version of your revised paper without tracked changes. You should upload this as a separate file labeled 'Manuscript'.

If you would like to make changes to your financial disclosure, please include your updated statement in your cover letter. Guidelines for resubmitting your figure files are available below the reviewer comments at the end of this letter.

If applicable, we recommend that you deposit your laboratory protocols in protocols.io to enhance the reproducibility of your results. Protocols.io assigns your protocol its own identifier (DOI) so that it can be cited independently in the future. For instructions see: https://journals.plos.org/plosone/s/submission-guidelines#loc-laboratory-protocols. Additionally, PLOS ONE offers an option for publishing peer-reviewed Lab Protocol articles, which describe protocols hosted on protocols.io. Read more information on sharing protocols at https://plos.org/protocols?utm_medium=editorial-email&utm_source=authorletters&utm_campaign=protocols.

We look forward to receiving your revised manuscript.

Kind regards,

Kangkang Ji, Ph.D., M.D.

Academic Editor

PLOS One

Response:

Dear Dr. Ji and Reviewers,

We sincerely thank you for the valuable time and insightful comments on our manuscript. We have carefully considered all the suggestions and have revised the manuscript accordingly. Below is a point-by-point response to each comment raised by the reviewers and the editorial office. All changes in the manuscript are highlighted in blue font for easy identification.

Response to Editorial Office Requirements

Journal Requirements:

When submitting your revision, we need you to address these additional requirements.

1. Please ensure that your manuscript meets PLOS ONE's style requirements, including those for file naming. The PLOS ONE style templates can be found at https://journals.plos.org/plosone/s/file?id=wjVg/PLOSOne_formatting_sample_main_body.pdf and https://journals.plos.org/plosone/s/file?id=ba62/PLOSOne_formatting_sample_title_authors_affiliations.pdf

Response: We have reviewed the PLOS ONE formatting templates and ensured that our manuscript meets the style requirements, including file naming and structure.

2. Please note that PLOS One has specific guidelines on code sharing for submissions in which author-generated code underpins the findings in the manuscript. In these cases, we expect all author-generated code to be made available without restrictions upon publication of the work. Please review our guidelines at https://journals.plos.org/plosone/s/materials-and-software-sharing#loc-sharing-code and ensure that your code is shared in a way that follows best practice and facilitates reproducibility and reuse.

Response: Our study did not generate custom code; all analyses were performed using publicly available tools as described in the Methods section. No code sharing is applicable.

3. Please update your submission to use the PLOS LaTeX template. The template and more information on our requirements for LaTeX submissions can be found at http://journals.plos.org/plosone/s/latex.

Response: Thank you for reminding us about the PLOS LaTeX template. We have carefully reviewed the guidelines at the provided link.

We would like to inform the editor that our revised manuscript has been prepared using Microsoft Word and has been carefully formatted to meet all PLOS ONE style requirements as outlined in the journal's formatting templates.

Given that our manuscript is fully prepared in Word and contains no complex mathematical or scientific notation that would necessitate LaTeX, we respectfully request permission to submit the manuscript in Word format. The manuscript file, figures, and supporting information are ready for submission as per the standard procedure. We believe this format will not compromise the review or production process. If the editor insists on a LaTeX submission, we will of course comply and undertake the necessary conversion.

Thank you for your understanding.

4. Thank you for stating the following in the Acknowledgments Section of your manuscript:

“This study was supported by the Natural Science Foundation of Ningxia (Grant No. [2022AAC03564])”

We note that you have provided funding information that is not currently declared in your Funding Statement. However, funding information should not appear in the Acknowledgments section or other areas of your manuscript. We will only publish funding information present in the Funding Statement section of the online submission form.

Please remove any funding-related text from the manuscript and let us know how you would like to update your Funding Statement. Currently, your Funding Statement reads as follows:

“This study was supported by the Natural Science Foundation of Ningxia (Grant No. [2022AAC03564]).”

Please include your amended statements within your cover letter; we will change the online submission form on your behalf.

Response:

We would like to respectfully clarify that in our submitted manuscript, the Acknowledgments section actually reads "None." , and the funding information was placed in a separate "Funding" section after the Acknowledgments, as is common practice in many journals.

Nevertheless, we understand and comply with PLOS ONE's policy that funding information should not appear in the manuscript text but should only be declared in the online submission system. Therefore, we have:

1.Removed the entire "Funding" section from the manuscript

2.Included the funding statement in our cover letter as requested

3.Confirmed that the online submission form will be updated accordingly with the following statement:

"This study was supported by the Natural Science Foundation of Ningxia (Grant No. [2022AAC03564]). The funders had no role in study design, data collection and analysis, decision to publish, or preparation of the manuscript."

We appreciate the editor's attention to this detail and have made the necessary corrections.

5. We note that the grant information you provided in the ‘Funding Information’ and ‘Financial Disclosure’ sections do not match.

When you resubmit, please ensure that you provide the correct grant numbers for the awards you received for your study in the ‘Funding Information’ section.

Response: We have verified that the grant information in the ‘Funding Information’ and ‘Financial Disclosure’ sections match. The correct grant number is [2022AAC03564].

6. Thank you for stating the following financial disclosure:

“This study was supported by the Natural Science Foundation of Ningxia (Grant No. [2022AAC03564]).”

Please state what role the funders took in the study.  If the funders had no role, please state: "The funders had no role in study design, data collection and analysis, decision to publish, or preparation of the manuscript."

If this statement is not correct you must amend it as needed.

Please include this amended Role of Funder statement in your cover letter; we will change the online submission form on your behalf.

Response: As stated above, we have included the role statement in the cover letter. The funders had no role in the study.

7. PLOS ONE now requires that authors provide the original uncropped and unadjusted images underlying all blot or gel results reported in a submission’s figures or Supporting Information files. This policy and the journal’s other requirements for blot/gel reporting and figure preparation are described in detail at https://journals.plos.org/plosone/s/figures#loc-blot-and-gel-reporting-requirements and https://journals.plos.org/plosone/s/figures#loc-preparing-figures-from-image-files. When you submit your revised manuscript, please ensure that your figures adhere fully to these guidelines and provide the original underlying images for all blot or gel data reported in your submission. See the following link for instructions on providing the original image data: https://journals.plos.org/plosone/s/figures#loc-original-images-for-blots-and-gels.

In your cover letter, please note whether your blot/gel image data are in Supporting Information or posted at a public data repository, provide the repository URL if relevant, and provide specific details as to which raw blot/gel images, if any, are not available. Email us at plosone@plos.org if you have any questions.

Response: All original uncropped and unadjusted images for western blots are provided in the Supporting Information file.

8. PLOS requires an ORCID iD for the corresponding author in Editorial Manager on papers submitted after December 6th, 2016. Please ensure that you have an ORCID iD and that it is validated in Editorial Manager. To do this, go to ‘Update my Information’ (in the upper left-hand corner of the main menu), and click on the Fetch/Validate link next to the ORCID field. This will take you to the ORCID site and allow you to create a new iD or authenticate a pre-existing iD in Editorial Manager.

Response: The corresponding author (Na Li) has validated her ORCID iD (0009-0007-3141-0124 ) in Editorial Manager.

9. If the reviewer comments include a recommendation to cite specific previously published works, please review and evaluate these publications to determine whether they are relevant and should be cited. There is no requirement to cite these works unless the editor has indicated otherwise.

Response: The reviewers did not specifically request citation of any particular published works, so no additional citations have been added.

[Note: HTML markup is below. Please do not edit.]

Reviewers' comments:

Reviewer's Responses to Questions

Comments to the Author

1. Is the manuscript technically sound, and do the data support the conclusions?

The manuscript must describe a technically sound piece of scientific research with data that supports the conclusions. Experiments must have been conducted rigorously, with appropriate controls, replication, and sample sizes. The conclusions must be drawn appropriately based on the data presented.

Reviewer #1: Yes

Reviewer #2: Yes

2. Has the statistical analysis been performed appropriately and rigorously?

Reviewer #1: I Don't Know

Reviewer #2: Yes

3. Have the authors made all data underlying the findings in their manuscript fully available?

The PLOS Data policy requires authors to make all data underlying the findings described in their manuscript fully available without restriction, with rare exception (please refer to the Data Availability Statement in the manuscript PDF file). The data should be provided as part of the manuscript or its supporting information, or deposited to a public repository. For example, in addition to summary statistics, the data points behind means, medians and variance measures should be available. If there are restrictions on publicly sharing data—e.g. participant privacy or use of data from a third party—those must be specified.

Reviewer #1: Yes

Reviewer #2: Yes

4. Is the manuscript presented in an intelligible fashion and written in standard English?

PLOS ONE does not copyedit accepted manuscripts, so the language in submitted articles must be clear, correct, and unambiguous. Any typographical or grammatical errors should be corrected at revision, so please note any specific errors here.

Reviewer #1: Yes

Reviewer #2: Yes

5. Review Comments to the Author

Please use the space provided to explain your answers to the questions above. You may also include additional comments for the author, including concerns about dual publication, research ethics, or publication ethics. (Please upload your review as an attachment if it exceeds 20,000 characters)

Reviewer #1: Overall this is a well-written paper that presents some important results. There are a few mostly minor things:

1) Western in western blotting should not be capitalized. Southern in Southern blotting is capitalized since the technique was invented by Dr. Southern. Northern blotting and western blotting were so named as a kind of scientific joke (not invented by a Dr. Northern and Dr. Western). This is not a rare error in scientific writing.

Response: We thank the reviewer for pointing out this common error. We have corrected “Western blot” to “western blot” throughout the manuscript. Page 4, Line 104; Page 9, Line 230; Page 17, Line 510.

2) In the Cell Proliferation assay, I would change the last sentence to say the "The net cell proliferation rate..." rather than "The cell proliferation rate..." since the CCK-8 assay results are proportional to the number of viable cells and therefore cannot distinguish whether there was just a change in in proliferation or if cell death was also involved.

Response: We agree with the reviewer’s suggestion. We have revised the description in manuscript to use “net cell proliferation rate” to reflect that CCK-8 measures viable cell number (Page 4, Line 113; Page 8, Line 215), which can be affected by both proliferation and cell death.

3) For evaluating RNA integrity before RNA sequencing, calculating an RNA integrity number would have been preferable to just using the A260/280 ration and (visually?) examining RNA gels.

Response: We thank the reviewer for this valuable suggestion. We fully acknowledge that the RNA Integrity Number (RIN) is currently the gold standard for assessing RNA quality, providing a more precise and objective measure of RNA integrity.

In our study, we assessed RNA integrity using A260/A280 ratios combined with agarose gel electrophoresis. The detailed quality control results for all six samples (three si-NC and three si-EIF1 biological replicates) are as follows:

Sample Sample Name Concentration(ng/ul) A260/A280 Volume(ul) Total Amount(ug) Overall Conclusion

1 ARPE-19 si-NC-1 174.24 1.995 9.5 1.66 Low risk

2 ARPE-19 si-NC-2 225.20 2.112 9.5 2.14 Qualified

3 ARPE-19 si-NC-3 231.80 2.056 9.5 2.20 Qualified

4 ARPE-19 si-EIF1-3-1 209.28 2.141 9.5 1.99 Low risk

5 ARPE-19 si-EIF1-3-2 227.12 2.008 9.5 2.16 Qualified

6 ARPE-19 si-EIF1-3-3 242.40 2.085 9.5 2.30 Qualified

As shown above, all samples exhibited A260/A280 ratios between 1.995 and 2.141, indicating high purity with minimal protein or organic solvent contamination. Additionally, agarose gel electrophoresis revealed clear, distinct ribosomal RNA bands (28S and 18S) with no visible degradation or smearing, and the bands were consistently observed in the 300-400 bp range, confirming that the RNA was intact and suitable for downstream library preparation and sequencing.

We agree with the reviewer that RIN provides a more standardized and quantitative measure of RNA integrity. Nevertheless, the combination of high A260/A280 ratios and clear gel electrophoresis results in this study provides sufficient evidence that the RNA quality was adequate for reliable RNA-seq analysis.

We appreciate the reviewer's attention to this important technical detail.

4) In the Statistical analysis section of Materials and methods, did the authors mean to say that the data was presented as the mean plus and mi

Attachment Submitted filename: Response to reviewers.docx

# Peer review: Decision Letter 1

**DOI:** 10.1371/journal.pone.0355473.r003

**Authors:** Hodaka Fujii

26 May 2026

Dear Dr. Li,

Please submit your revised manuscript by Jul 10 2026 11:59PM. If you will need more time than this to complete your revisions, please reply to this message or contact the journal office at plosone@plos.org. When you're ready to submit your revision, log on to https://www.editorialmanager.com/pone/ and select the 'Submissions Needing Revision' folder to locate your manuscript file.

- A letter that responds to each point raised by the academic editor and reviewer(s). You should upload this letter as a separate file labeled 'Response to Reviewers'.
- A marked-up copy of your manuscript that highlights changes made to the original version. You should upload this as a separate file labeled 'Revised Manuscript with Track Changes'.
- An unmarked version of your revised paper without tracked changes. You should upload this as a separate file labeled 'Manuscript'.

If you would like to make changes to your financial disclosure, please include your updated statement in your cover letter. Guidelines for resubmitting your figure files are available below the reviewer comments at the end of this letter.

If applicable, we recommend that you deposit your laboratory protocols in protocols.io to enhance the reproducibility of your results. Protocols.io assigns your protocol its own identifier (DOI) so that it can be cited independently in the future. For instructions see: https://journals.plos.org/plosone/s/submission-guidelines#loc-laboratory-protocols. Additionally, PLOS ONE offers an option for publishing peer-reviewed Lab Protocol articles, which describe protocols hosted on protocols.io. Read more information on sharing protocols at https://plos.org/protocols?utm_medium=editorial-email&utm_source=authorletters&utm_campaign=protocols.

As the corresponding author, your ORCID iD is verified in the submission system and will appear in the published article. PLOS supports the use of ORCID, and we encourage all coauthors to register for an ORCID iD and use it as well. Please encourage your coauthors to verify their ORCID iD within the submission system before final acceptance, as unverified ORCID iDs will not appear in the published article. *Only*  the individual author can complete the verification step; PLOS staff *cannot*  verify ORCID iDs on behalf of authors.

We look forward to receiving your revised manuscript.

Kind regards,

Hodaka Fujii, M.D., Ph.D.

Academic Editor

PLOS One

Journal Requirements:

If the reviewer comments include a recommendation to cite specific previously published works, please review and evaluate these publications to determine whether they are relevant and should be cited. There is no requirement to cite these works unless the editor has indicated otherwise.

[Note: HTML markup is below. Please do not edit.]

Reviewers' comments:

Reviewer's Responses to Questions

**Comments to the Author**

Reviewer #1: All comments have been addressed

Reviewer #2: (No Response)

Reviewer #3: (No Response)

Reviewer #4: All comments have been addressed

**********

2. Is the manuscript technically sound, and do the data support the conclusions?

Reviewer #1: Yes

Reviewer #2: Partly

Reviewer #3: Yes

Reviewer #4: Yes

**********

3. Has the statistical analysis been performed appropriately and rigorously?

Reviewer #1: Yes

Reviewer #2: Yes

Reviewer #3: Yes

Reviewer #4: Yes

**********

4. Have the authors made all data underlying the findings in their manuscript fully available?

The PLOS Data policy

Reviewer #1: Yes

Reviewer #2: Yes

Reviewer #3: Yes

Reviewer #4: Yes

**********

5. Is the manuscript presented in an intelligible fashion and written in standard English?

Reviewer #1: Yes

Reviewer #2: Yes

Reviewer #3: Yes

Reviewer #4: No

**********

Reviewer #1: This manuscript is significantly improved over the original submission. Changes have been made that clarify information in the Materials and Methods and in the Results and the Discussion that address the limitations of the study.

The English is good, although not perfect. Please note that the first sentence of the Discussion (line 299) is not a good sentence. I suggest removing the word "is" after "DR".

Note: Most writing by native English speakers is not perfect. Also, the use of articles in language can be tricky. Chinese does not use them, English does but uses them much less than French while Russian also uses articles, but uses them much less than English does.

Overall, I think that this work provides some significant information that will be useful in furthering the understanding and potentially treatment of DR

Reviewer #2: The revision corrects most Round 1 deficiencies. RT-qPCR confirmation of differential splicing in COP1 and TFE3 provides the experimental validation the splicing analysis required, and moving MCM7, CPSF4, and HMGA1 to supplementary figures is the correct approach given that they could not be confirmed. Statistical reporting now distinguishes SD for cellular assays from SEM for RNA-seq-derived FPKM values, with Tukey's post-hoc test specified for two-way ANOVA comparisons. Biological replication (three independent cultures per condition, sequenced in a single run) is now stated. The ARPE-19 limitation, osmotic confound from 50 mM glucose without mannitol control, and absence of functional validation are now explicitly acknowledged in the limitations section. The abstract and most of the body use appropriately hedged language framing the findings as associative and hypothesis-generating. Two concerns remain.

1. (Major) Raw RNA-seq reads have not been deposited in a public repository. The Data Availability Statement currently reads "All relevant data are within the manuscript and its Supporting Information files." Primary sequencing reads cannot be embedded in a manuscript; PLOS ONE data policy requires deposition in NCBI GEO or SRA prior to acceptance, with the accession number provided in the Data Availability Statement and cited in the Methods. No GEO or SRA accession number appears anywhere in the submission. The authors must deposit the raw reads, generate an accession number, and update the Data Availability Statement and Methods accordingly before this manuscript can be accepted.

2. (Minor) The Discussion closing passages retain overclaiming language that was not corrected. Line 331 places EIF1 as "a pivotal molecular mediator linking hyperglycemia to cell cycle dysregulation in DR." Line 367 opens the limitations paragraph with "Despite the breakthrough implications of our findings for DR therapeutics." Lines 387-391 state the work "elucidates pivotal connections" and holds "substantial translational value...offering innovative perspectives that could reshape future treatment paradigms." These passages are inconsistent with the moderated abstract and with a study constrained to a single transformed cell line, lacking an osmotic control, and providing no causal functional evidence. The language should be brought to the same epistemic level as the rest of the revised manuscript. The same section also contains a grammatical error at line 295-296 ("mechanistically may implicating their dysregulation") that persists despite the stated language revision.

Reviewer #3: This study investigates the role of eukaryotic translation initiation factor 1 (EIF1) in diabetic retinopathy (DR). Using an in vitro model of high glucose-treated human retinal pigment epithelial cells (ARPE-19), we observed that high glucose levels significantly inhibited cell proliferation, a phenomenon associated with EIF1 downregulation. This suggests that EIF1 depletion induced by high glucose may impair cellular function. Transcriptome sequencing revealed that EIF1 knockdown co-induced 223 differentially expressed genes, with 188 of these downregulated genes being significantly enriched in cell cycle-related pathways. Concurrently, 1211 differential alternative splicing events were detected, primarily impacting the cell cycle and p53 signaling pathway. The key differentially expressed genes and splicing pattern changes of COP1 and TFE3 were validated using RT-qPCR. These findings suggest that EIF1 may contribute to cell cycle dysregulation in DR by regulating gene expression and alternative splicing, thereby laying the groundwork for future mechanistic investigations.

1. In the abstract and introduction sections, it is recommended to further clarify the distinction between EIF1's roles in translation initiation and RNA binding functions to avoid conflating these two processes.

2. The following references are closely related to the authors' research topic and should be cited:

[1] Yan Z-H, Zhao D-M, Wang X-T, et al. “Medicine food homology” plants in the treatment of diabetic nephropathy: pathogenic pathways and therapeutic approaches. Food & Medicine Homology, 2025, https://doi.org/10.26599/FMH.2026.9420115

[2] Asare, A., Adjei Broni, A., Asare Dickson, A.K., Sagoe, M. and Cudjoe, J.M. (2025), Performance of ResNet-18 and InceptionResNetV2 in Automated Detection of Diabetic Retinopathy. Med. Adv, 3: 231-241. https://doi.org/10.1002/med4.70023

[3] Li M-Y, Wu G-F, Lu F, et al. Food and Medicine Homology Focus in 2026. Food & Medicine Homology, 2026, 3(2): 9420133. https://doi.org/10.26599/FMH.2026.9420133

3. The description of cell cycle-related genes in the discussion section appears overly list-like; it is suggested to strengthen the functional connections among these genes and their synergistic effects in differentiation-related (DR) processes.

4. For unverified alternative splicing (AS) events, potential technical reasons should be briefly explained in the main text.

5. In the conclusion section, strong statements such as "providing theoretical support for precise treatment" should be revised to more moderate expressions like "laying a foundation for subsequent studies" or "suggesting their potential value."

6. In the discussion section, the mechanism by which EIF1 simultaneously regulates transcription and splicing needs clarification regarding whether this involves direct or indirect actions, to prevent mechanistic confusion when presenting these two functions side by side.

7. For genes with validated splicing events such as COP1 and TFE3, the possible functional differences of their splice variants should be briefly discussed in the main text to enhance the logical connection with cell cycle regulation.

8. It is recommended to standardize the terminology for "cell cycle dysregulation" throughout the manuscript, avoiding inconsistent use of terms like "abnormal," "disordered," or "dysregulated" in different chapters to improve terminological consistency.

Reviewer #4: Reviewer Comments

This study focuses on the molecular mechanism of EIF1 regulating the cell cycle in diabetic retinopathy (DR) using a classic research approach combining RNA-seq with functional validation. The research topic aligns with PLOS ONE’s focus on basic medical and molecular mechanism research. The experimental design is basically rational, the data are complete, and most of the requirements for the first round of revision have been met.

1. General Evaluation

Using ARPE-19 cells as an in vitro model, this study investigated the expression changes and functions of EIF1 in high glucose-induced DR, and found that EIF1 knockdown leads to cell cycle dysregulation by regulating the differential expression and alternative splicing of cell cycle-related genes. It is the first to reveal the potential role of EIF1 in the functional abnormalities of retinal pigment epithelial cells in DR, providing new clues for the study of the molecular mechanism of DR. The authors have completed key revisions in accordance with the first round of reviewer comments, including weakening causal conclusions, supplementing experimental validation, improving methodological details, and expanding research limitations, which have greatly enhanced the scientificity and rigor of the manuscript. At present, the manuscript still has some minor details to be further improved, with no defects in core scientific issues, and it is recommended to accept after minor revision.

2.Specific Revision Suggestions

(1)Supplementary Methodological Details

Description of cell experimental groups: In 2.3 Cell proliferation assay, only 0, 30, and 50 mM glucose groups are mentioned, and the glucose concentration of the "normal control group" (routinely 5.5 mM) is not specified. It is recommended to supplement this basic information to standardize the experimental grouping and facilitate readers to understand the basis for setting the concentration gradient of high glucose treatment.

(2)RT-qPCR primer information: The manuscript mentions that primer information is shown in Supplementary Table S1, but it is not clearly stated in the manuscript whether Table S1 contains core information such as primer sequences, amplified fragment lengths, and annealing temperatures. It is recommended to confirm the completeness of this information in the description of supplementary materials to meet the repeatability requirements of molecular experiments.

(3)Quantification description of protein detection: Section 2.5 Western blot only describes the experimental procedure, without specifying the quantification method (e.g., gray value analysis) and software used for protein bands. It is recommended to supplement this detail to make the analysis of protein experimental results more operable.

3.Optimization of Result Presentation and Figures/Tables

(1)Consistency of result data presentation: There are inconsistent formats of statistical significance labels in some results (e.g., ****p<0.0001 in Fig 1, and p<0.0001 in some main text). It is recommended to unify the format of statistical significance labels throughout the manuscript (including figure legends and main text) to maintain standardization.

(2)Simplification of figure legends and labels: The annotation of enrichment analysis bubble plots in some supplementary figures (e.g., Fig S2) is slightly dense, and the font of some pathway names is too small. It is recommended to appropriately simplify the legend annotation and enlarge the core pathway names to improve the readability of the figures; at the same time, confirm that the sample size (n value) of all figures is clearly marked in the figure legends without omission.

(3)Presentation of alternative splicing results: Section 3.3 mentions that 5 alternative splicing-related genes were screened out, and only COP1 and TFE3 were validated, with the remaining 3 moved to supplementary materials. It is recommended to mark in the main text that "the splicing changes of other candidate genes were not verified by experiments, see Supplementary Materials" to make the result presentation more rigorous and avoid misunderstanding by readers.

4.Improvement of Discussion and Conclusions

(1)Appropriate elaboration of research significance: The discussion section has fully explained the research limitations, and the preliminary value of this study can be appropriately supplemented, such as "This study is the first to associate EIF1 with the cell cycle regulation of retinal pigment epithelial cells in DR, providing candidate molecules and transcriptomic data support for the subsequent exploration of new targets for DR", to achieve a better balance in the discussion and avoid the research significance being less prominent due to overemphasis on limitations.

(2)Optimization of the conciseness of conclusions: Section 5 Conclusions is slightly lengthy and can be appropriately streamlined to highlight the core findings (EIF1 is downregulated under high glucose, regulates the expression and splicing of cell cycle-related genes, and provides new candidates for DR research), delete repetitive expressions, and make the conclusions more concise in line with PLOS ONE’s writing requirements for clarity and conciseness.

5.Correction of Format and Text Details

(1)English spelling and punctuation: Check the English spelling and punctuation throughout the manuscript, e.g., "Refferences" should be corrected to "References", and the punctuation separation (e.g., commas and semicolons) in some long sentences needs to be further standardized to avoid affecting the manuscript quality due to minor format errors.

(2)First annotation of abbreviations: Some abbreviations in the manuscript (e.g., ASEs, RASEs, RASGs) have been annotated at their first occurrence, but it is recommended to supplement the full spelling at their first occurrence in the Abstract (e.g., alternative splicing events, ASEs) to facilitate independent reading of the abstract.

(3)Reference format: Check the consistency of reference formats, e.g., different annotation methods for volume, issue and page numbers of some literatures. It is recommended to unify the format in accordance with PLOS ONE’s reference requirements to ensure compliance with the journal typesetting specifications.

**********

what does this mean?). If published, this will include your full peer review and any attached files.

If you choose “no”, your identity will remain anonymous but your review may still be made public.

**Do you want your identity to be public for this peer review?**  For information about this choice, including consent withdrawal, please see our Privacy Policy

Reviewer #1: No

Reviewer #2: No

Reviewer #3: No

Reviewer #4: No

**********

[NOTE: If reviewer comments were submitted as an attachment file, they will be attached to this email and accessible via the submission site. Please log into your account, locate the manuscript record, and check for the action link "View Attachments". If this link does not appear, there are no attachment files.]

To ensure your figures meet our technical requirements, please review our figure guidelines: https://journals.plos.org/plosone/s/figures

You may also use PLOS’s free figure tool, NAAS, to help you prepare publication quality figures: https://journals.plos.org/plosone/s/figures#loc-tools-for-figure-preparation.

NAAS will assess whether your figures meet our technical requirements by comparing each figure against our figure specifications.

# Author comment: Author response to Decision Letter 2

**DOI:** 10.1371/journal.pone.0355473.r004

26 Jun 2026

Dear Dr. Li,

Thank you for submitting your manuscript to PLOS ONE. After careful consideration, we feel that it has merit but does not fully meet PLOS ONE’s publication criteria as it currently stands. Therefore, we invite you to submit a revised version of the manuscript that addresses the points raised during the review process.

Your revised manuscript was reviewed by the four referees who had reviewed the original one. Although they found it significantly improved, they still raised some concerns. Please revise it according to their suggestions.

Please submit your revised manuscript by Jul 10 2026 11:59PM. If you will need more time than this to complete your revisions, please reply to this message or contact the journal office at plosone@plos.org. When you're ready to submit your revision, log on to https://www.editorialmanager.com/pone/ and select the 'Submissions Needing Revision' folder to locate your manuscript file.

Please include the following items when submitting your revised manuscript:

A letter that responds to each point raised by the academic editor and reviewer(s). You should upload this letter as a separate file labeled 'Response to Reviewers'.

A marked-up copy of your manuscript that highlights changes made to the original version. You should upload this as a separate file labeled 'Revised Manuscript with Track Changes'.

An unmarked version of your revised paper without tracked changes. You should upload this as a separate file labeled 'Manuscript'.

If you would like to make changes to your financial disclosure, please include your updated statement in your cover letter. Guidelines for resubmitting your figure files are available below the reviewer comments at the end of this letter.

If applicable, we recommend that you deposit your laboratory protocols in protocols.io to enhance the reproducibility of your results. Protocols.io assigns your protocol its own identifier (DOI) so that it can be cited independently in the future. For instructions see: https://journals.plos.org/plosone/s/submission-guidelines#loc-laboratory-protocols. Additionally, PLOS ONE offers an option for publishing peer-reviewed Lab Protocol articles, which describe protocols hosted on protocols.io. Read more information on sharing protocols at https://plos.org/protocols?utm_medium=editorial-email&utm_source=authorletters&utm_campaign=protocols.

As the corresponding author, your ORCID iD is verified in the submission system and will appear in the published article. PLOS supports the use of ORCID, and we encourage all coauthors to register for an ORCID iD and use it as well. Please encourage your coauthors to verify their ORCID iD within the submission system before final acceptance, as unverified ORCID iDs will not appear in the published article. Only the individual author can complete the verification step; PLOS staff cannot verify ORCID iDs on behalf of authors.

We look forward to receiving your revised manuscript.

Kind regards,

Hodaka Fujii, M.D., Ph.D.

Academic Editor

PLOS One

Journal Requirements:

If the reviewer comments include a recommendation to cite specific previously published works, please review and evaluate these publications to determine whether they are relevant and should be cited. There is no requirement to cite these works unless the editor has indicated otherwise.

[Note: HTML markup is below. Please do not edit.]

Dear Dr. Fujii and Reviewers,

We thank the academic editor and all reviewers for their constructive comments on our manuscript (PONE-D-25-49013R1). We have carefully addressed each point raised. Below we provide point-by-point responses. The corresponding revisions have been made in the marked-up copy of the manuscript (submitted as “Revised Manuscript with Track Changes”) and are also reflected in the clean version (“Manuscript”).

Reviewers' comments:

Reviewer's Responses to Questions

Comments to the Author

1. If the authors have adequately addressed your comments raised in a previous round of review and you feel that this manuscript is now acceptable for publication, you may indicate that here to bypass the “Comments to the Author” section, enter your conflict of interest statement in the “Confidential to Editor” section, and submit your "Accept" recommendation.

Reviewer #1: All comments have been addressed

Reviewer #2: (No Response)

Reviewer #3: (No Response)

Reviewer #4: All comments have been addressed

2. Is the manuscript technically sound, and do the data support the conclusions?

The manuscript must describe a technically sound piece of scientific research with data that supports the conclusions. Experiments must have been conducted rigorously, with appropriate controls, replication, and sample sizes. The conclusions must be drawn appropriately based on the data presented.

Reviewer #1: Yes

Reviewer #2: Partly

Reviewer #3: Yes

Reviewer #4: Yes

3. Has the statistical analysis been performed appropriately and rigorously?

Reviewer #1: Yes

Reviewer #2: Yes

Reviewer #3: Yes

Reviewer #4: Yes

4. Have the authors made all data underlying the findings in their manuscript fully available?

The PLOS Data policy requires authors to make all data underlying the findings described in their manuscript fully available without restriction, with rare exception (please refer to the Data Availability Statement in the manuscript PDF file). The data should be provided as part of the manuscript or its supporting information, or deposited to a public repository. For example, in addition to summary statistics, the data points behind means, medians and variance measures should be available. If there are restrictions on publicly sharing data—e.g. participant privacy or use of data from a third party—those must be specified.

Reviewer #1: Yes

Reviewer #2: Yes

Reviewer #3: Yes

Reviewer #4: Yes

5. Is the manuscript presented in an intelligible fashion and written in standard English?

PLOS ONE does not copyedit accepted manuscripts, so the language in submitted articles must be clear, correct, and unambiguous. Any typographical or grammatical errors should be corrected at revision, so please note any specific errors here.

Reviewer #1: Yes

Reviewer #2: Yes

Reviewer #3: Yes

Reviewer #4: No

6. Review Comments to the Author

Please use the space provided to explain your answers to the questions above. You may also include additional comments for the author, including concerns about dual publication, research ethics, or publication ethics. (Please upload your review as an attachment if it exceeds 20,000 characters)

Reviewer #1: This manuscript is significantly improved over the original submission. Changes have been made that clarify information in the Materials and Methods and in the Results and the Discussion that address the limitations of the study.

The English is good, although not perfect. Please note that the first sentence of the Discussion (line 299) is not a good sentence. I suggest removing the word "is" after "DR".

Note: Most writing by native English speakers is not perfect. Also, the use of articles in language can be tricky. Chinese does not use them, English does but uses them much less than French while Russian also uses articles, but uses them much less than English does.

Overall, I think that this work provides some significant information that will be useful in furthering the understanding and potentially treatment of DR.

Response: We are grateful for this careful reading. We have corrected the first sentence of the Discussion as recommended. The original sentence “DR is one of the most severe microvascular complications of diabetes mellitus, remains incompletely understood…” has been revised to “DR, one of the most severe microvascular complications of diabetes mellitus, remains incompletely understood…” (Line 312). Thank you again for improving our manuscript.

Reviewer #2: The revision corrects most Round 1 deficiencies. RT-qPCR confirmation of differential splicing in COP1 and TFE3 provides the experimental validation the splicing analysis required, and moving MCM7, CPSF4, and HMGA1 to supplementary figures is the correct approach given that they could not be confirmed. Statistical reporting now distinguishes SD for cellular assays from SEM for RNA-seq-derived FPKM values, with Tukey's post-hoc test specified for two-way ANOVA comparisons. Biological replication (three independent cultures per condition, sequenced in a single run) is now stated. The ARPE-19 limitation, osmotic confound from 50 mM glucose without mannitol control, and absence of functional validation are now explicitly acknowledged in the limitations section. The abstract and most of the body use appropriately hedged language framing the findings as associative and hypothesis-generating. Two concerns remain.

1.(Major) Raw RNA-seq reads have not been deposited in a public repository. The Data Availability Statement currently reads "All relevant data are within the manuscript and its Supporting Information files." Primary sequencing reads cannot be embedded in a manuscript; PLOS ONE data policy requires deposition in NCBI GEO or SRA prior to acceptance, with the accession number provided in the Data Availability Statement and cited in the Methods. No GEO or SRA accession number appears anywhere in the submission. The authors must deposit the raw reads, generate an accession number, and update the Data Availability Statement and Methods accordingly before this manuscript can be accepted.

Response: We thank the reviewer for pointing out this critical omission. We have now deposited the raw RNA-seq reads in the NCBI Gene Expression Omnibus (GEO) under accession number GSE334496. We have updated the Data Availability Statement in the manuscript. The revised Data Availability Statement reads (Line 415-417): “The raw RNA-seq data generated in this study have been deposited in the NCBI Gene Expression Omnibus (GEO) under accession number GSE334496.” We are grateful for this essential guidance.

2. (Minor) The Discussion closing passages retain overclaiming language that was not corrected. Line 331 places EIF1 as "a pivotal molecular mediator linking hyperglycemia to cell cycle dysregulation in DR." Line 367 opens the limitations paragraph with "Despite the breakthrough implications of our findings for DR therapeutics." Lines 387-391 state the work "elucidates pivotal connections" and holds "substantial translational value...offering innovative perspectives that could reshape future treatment paradigms." These passages are inconsistent with the moderated abstract and with a study constrained to a single transformed cell line, lacking an osmotic control, and providing no causal functional evidence. The language should be brought to the same epistemic level as the rest of the revised manuscript. The same section also contains a grammatical error at line 295-296 ("mechanistically may implicating their dysregulation") that persists despite the stated language revision.

Response: We thank the reviewer for this important reminder to maintain appropriate scientific caution. We have toned down the overclaiming language throughout the Discussion and Conclusion sections. Specific changes are as follows:

•Line 343-345: “These results position EIF1 as a pivotal molecular mediator linking hyperglycemia to cell cycle dysregulation in DR” → Revised: “These results suggest that EIF1 may be associated with cell cycle dysregulation in DR under hyperglycemic conditions”.

•Line 383-384: “Despite the breakthrough implications of our findings for DR therapeutics” → Revised: “Although our findings suggest potential relevance for understanding DR pathogenesis”.

•Grammatical error (Line 308): “mechanistically may implicating their dysregulation” → Revised: “potentially implicating their dysregulation”.

We are grateful for the reviewer’s meticulous attention to language.

Reviewer #3: This study investigates the role of eukaryotic translation initiation factor 1 (EIF1) in diabetic retinopathy (DR). Using an in vitro model of high glucose-treated human retinal pigment epithelial cells (ARPE-19), we observed that high glucose levels significantly inhibited cell proliferation, a phenomenon associated with EIF1 downregulation. This suggests that EIF1 depletion induced by high glucose may impair cellular function. Transcriptome sequencing revealed that EIF1 knockdown co-induced 223 differentially expressed genes, with 188 of these downregulated genes being significantly enriched in cell cycle-related pathways. Concurrently, 1211 differential alternative splicing events were detected, primarily impacting the cell cycle and p53 signaling pathway. The key differentially expressed genes and splicing pattern changes of COP1 and TFE3 were validated using RT-qPCR. These findings suggest that EIF1 may contribute to cell cycle dysregulation in DR by regulating gene expression and alternative splicing, thereby laying the groundwork for future mechanistic investigations.

1.In the abstract and introduction sections, it is recommended to further clarify the distinction between EIF1's roles in translation initiation and RNA binding functions to avoid conflating these two processes.

Response: We thank the reviewer for this important suggestion to avoid conflating EIF1’s distinct functional roles. In response, we have revised the Introduction section to more clearly distinguish between EIF1’s canonical function in translation initiation and its capacity as an RNA-binding protein. The revised text now reads (Line 71-78): “Eukaryotic translation initiation factor 1 (EIF1; aliases: A121, ISO1, SUI1, EIF-1, EIF1A), a 12-kDa protein that functions as a critical mediator of start codon recognition during eukaryotic translation initiation[7-8]. Beyond its canonical role in translation initiation[9], EIF1 has been classified as an RNA-binding protein (RBP) based on its ability to interact with RNA molecules, and emerging evidence suggests that it may participate in...” This revision first establishes EIF1’s primary functional identity as a translation initiation factor, then separately introduces its RNA-binding capacity as a basis for potential broader regulatory roles, thus drawing a clear conceptual distinction between these two aspects. We are grateful for the reviewer’s careful reading and constructive suggestion.

2. The following references are closely related to the authors' research topic and should be cited:

[1] Yan Z-H, Zhao D-M, Wang X-T, et al. “Medicine food homology” plants in the treatment of diabetic nephropathy: pathogenic pathways and therapeutic approaches. Food & Medicine Homology, 2025, https://doi.org/10.26599/FMH.2026.9420115

[2] Asare, A., Adjei Broni, A., Asare Dickson, A.K., Sagoe, M. and Cudjoe, J.M. (2025), Performance of ResNet-18 and InceptionResNetV2 in Automated Detection of Diabetic Retinopathy. Med. Adv, 3: 231-241. https://doi.org/10.1002/med4.70023

[3] Li M-Y, Wu G-F, Lu F, et al. Food and Medicine Homology Focus in 2026. Food & Medicine Homology, 2026, 3(2): 9420133. https://doi.org/10.26599/FMH.2026.9420133

Response: We thank the reviewer for providing these references, which we have carefully reviewed.

Regarding references [1] and [3]: After thorough evaluation, we note that reference [1] focuses on diabetic nephropathy (not diabetic retinopathy) and the therapeutic application of “medicine-food homology” plants, while reference [3] is a perspective article on the field of food and medicine homology. Neither directly addresses the molecular mechanisms of diabetic retinopathy or the role of RNA-binding proteins such as EIF1 in this disease. Therefore, we respectfully conclude that references [1] and [3] are not directly relevant to our study and will not be cited.

Regarding reference [2] (Asare A et al., Performance of ResNet-18 and InceptionResNetV2 in Automated Detection of Diabetic Retinopathy, Med. Adv, 2025, 3: 231-241): This paper provides valuable background on the epidemiology of diabetic retinopathy (DR), noting that DR affects approximately 34.6% of diabetic individuals and is a leading cause of vision loss. Our manuscript presents similar epidemiological data (30–40% prevalence, over 100 million affected globally). Therefore, we have added reference [2] as an additional citation to support the existing

Attachment Submitted filename: Response_to_Reviewers_auresp_2.docx

# Peer review: Decision Letter 2

**DOI:** 10.1371/journal.pone.0355473.r005

**Authors:** Hodaka Fujii

22 Jul 2026

EIF1 coordinates transcriptomic and splicing networks associated with cell cycle dysregulation in diabetic retinopathy

PONE-D-25-49013R2

Dear Dr. Li,

We’re pleased to inform you that your manuscript has been judged scientifically suitable for publication and will be formally accepted for publication once it meets all outstanding technical requirements.

Within one week, you’ll receive an e-mail detailing the required amendments. When these have been addressed, you’ll receive a formal acceptance letter and your manuscript will be scheduled for publication.

An invoice will be generated when your article is formally accepted. Please note, if your institution has a publishing partnership with PLOS and your article meets the relevant criteria, all or part of your publication costs will be covered. Please make sure your user information is up-to-date by logging into Editorial Manager at Editorial Manager® and clicking the ‘Update My Information' link at the top of the page. For questions related to billing, please contact billing support.

If your institution or institutions have a press office, please notify them about your upcoming paper to help maximize its impact. If they’ll be preparing press materials, please inform our press team as soon as possible -- no later than 48 hours after receiving the formal acceptance. Your manuscript will remain under strict press embargo until 2 pm Eastern Time on the date of publication. For more information, please contact onepress@plos.org.

Kind regards,

Hodaka Fujii, M.D., Ph.D.

Academic Editor

PLOS One

Additional Editor Comments (optional):

Reviewers' comments:

Reviewer's Responses to Questions

**Comments to the Author**

Reviewer #1: All comments have been addressed

Reviewer #4: All comments have been addressed

**********

2. Is the manuscript technically sound, and do the data support the conclusions?

Reviewer #1: Yes

Reviewer #4: Yes

**********

3. Has the statistical analysis been performed appropriately and rigorously?

Reviewer #1: Yes

Reviewer #4: Yes

**********

4. Have the authors made all data underlying the findings in their manuscript fully available?

The PLOS Data policy

Reviewer #1: Yes

Reviewer #4: Yes

**********

5. Is the manuscript presented in an intelligible fashion and written in standard English?

Reviewer #1: Yes

Reviewer #4: Yes

**********

Reviewer #1: (No Response)

Reviewer #4: The quality of this version of the manuscript has been improved and is recommended for publication.Thank you!

**********

what does this mean?). If published, this will include your full peer review and any attached files.

If you choose “no”, your identity will remain anonymous but your review may still be made public.

**Do you want your identity to be public for this peer review?**  For information about this choice, including consent withdrawal, please see our Privacy Policy

Reviewer #1: No

Reviewer #4: No

**********

# Editor report: Acceptance letter

**DOI:** 10.1371/journal.pone.0355473.r006

**Authors:** Hodaka Fujii

PONE-D-25-49013R2

PLOS One

Dear Dr. Li,

I'm pleased to inform you that your manuscript has been deemed suitable for publication in PLOS One. Congratulations! Your manuscript is now being handed over to our production team.

At this stage, our production department will prepare your paper for publication. This includes ensuring the following:

* All references, tables, and figures are properly cited

* All relevant supporting information is included in the manuscript submission,

* There are no issues that prevent the paper from being properly typeset

You will receive further instructions from the production team, including instructions on how to review your proof when it is ready. Please keep in mind that we are working through a large volume of accepted articles, so please give us a few days to review your paper and let you know the next and final steps.

Lastly, if your institution or institutions have a press office, please let them know about your upcoming paper now to help maximize its impact. If they'll be preparing press materials, please inform our press team within the next 48 hours. Your manuscript will remain under strict press embargo until 2 pm Eastern Time on the date of publication. For more information, please contact onepress@plos.org.

You will receive an invoice from PLOS for your publication fee after your manuscript has reached the completed accept phase. If you receive an email requesting payment before acceptance or for any other service, this may be a phishing scheme. Learn how to identify phishing emails and protect your accounts at https://explore.plos.org/phishing.

If we can help with anything else, please email us at customercare@plos.org.

Thank you for submitting your work to PLOS One and supporting open access.

Kind regards,

PLOS One Editorial Office Staff

on behalf of

Dr. Hodaka Fujii

Academic Editor

PLOS One
